# Regulation of cellulase production via calcium signaling in *Trichoderma reesei* under PEG8000 stress

**DOI:** 10.1007/s00253-023-12901-w

**Published:** 2024-01-26

**Authors:** Shuai Liu, Lin Quan, Mei Yang, Dan Wang, Yong-Zhong Wang

**Affiliations:** 1 Key Laboratory of Biorheological Science and Technology (Chongqing University), Ministry of Education, College of Bioengineering, Chongqing University, Chongqing, 400030 China; 2 School of Chemistry and Chemical Engineering, Chongqing University, Chongqing, 400044 China

**Keywords:** *Trichoderma reesei* CICC2626, Polyethylene glycol 8000, Cellulase production, Calcium signaling

## Abstract

**Abstract:**

In this study, the effect of polyethylene glycol 8000 (PEG8000) stress on cellulase biosynthesis in *Trichoderma reesei* CICC2626 via calcium signaling was investigated, and a plausible mechanism by which intracellular Ca^2+^ regulates the transcription of cellulase genes was proposed. The results indicated that the total cellulase (filter paper-hydrolyzing activity [FPase]), endoglucanase (carboxymethyl cellulase activity [CMCase]), and β-glucosidase activities of the strain were 1.3-, 1.2-, and 1.3-fold higher than those of the control (no PEG8000 addition) at a final concentration of 1.5% (w/v) PEG8000. Moreover, the transcriptional levels of cellulase genes, protein concentrations, and biomass increased. With the synergistic use of commercial cellulase and *T. reesei* CICC2626 cellulase to hydrolyze alkali-pretreated rice straw, the released reducing sugar concentration reached 372.7 mg/g, and the cellulose content (22.7%, 0.32 g) was significantly lower than the initial content (62.5%, 1.88 g). Transcriptome data showed that 12 lignocellulose degradation–related genes were significantly upregulated in the presence of 1.5% PEG8000. Furthermore, the addition of Ca^2+^ inhibitors and deletion of *crz1* (calcineurin-responsive zinc finger 1-encoding gene, which is related to the calcium signaling pathway) demonstrated that calcium signaling plays a dominant role in PEG8000-induced cellulase genes overexpression. These results revealed a link between PEG8000 induction and calcium signaling transduction in *T. reesei* CICC2626. Moreover, this study also provides a novel inducer for enhanced cellulase production.

**Key points:**

• *Cellulase biosynthesis in Trichoderma reesei could be enhanced by PEG8000*

• *PEG8000 could induce a cytosolic Ca*^*2+*^* burst in Trichoderma reesei*

• *The activated calcium signaling was involved in cellulase biosynthesis*

**Graphical abstract:**

**Figure:**
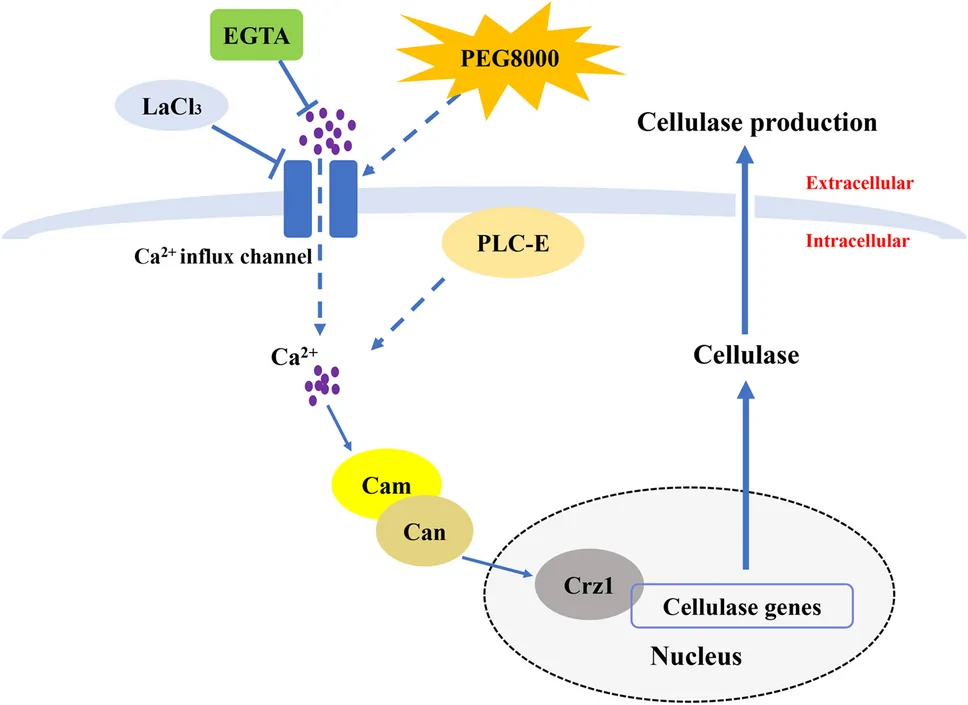

**Supplementary Information:**

The online version contains supplementary material available at 10.1007/s00253-023-12901-w.

## Introduction

In most developing countries, agricultural residues are usually disposed of in landfills, which can lead to serious environmental problems such as groundwater pollution. Residues such as straw, composed mainly of cellulose, hemicellulose, and lignin, are promising feedstocks for converting biofuels and other chemicals (Douvartzides et al. 2022). However, as a component of lignocellulosic substrates, lignin prevents cellulase from binding to cellulose, resulting in the difficult hydrolysis of natural cellulose (Saini et al. 2016). High cellulase activity is required during enzymatic saccharification to effectively convert cellulose into fermentable sugars. However, the low yield of cellulases is a crucial technical bottleneck for commercial utilization (Hao et al. 2016). As one of the most prominent cellulase producers in nature, *Trichoderma reesei* has been used to produce cellulase because of its high growth rate and ability to produce highly stable enzymes (Xu et al. 2017).

Additionally, cellulase production requires inducers, including cellulose (Derntl et al. 2017), cellobiose (Kubicek et al. 2009), and sophorose (Mandels et al. 1962), which have long been known. Therefore, it is necessary to identify novel inducers with high cellulase yield. Polyethylene glycol (PEG) is a flexible nonionic surfactant with a glycol subunit (HOCH_2_CH_2_OH) that induces hyperosmosis in the solution (Yang et al. 2014). Hemansi et al. (2018) found that the cellulase activity in *Aspergillus niger* significantly increased after supplementation with PEG8000. Liu et al. (2019) reported that the cellulase activity of *Bacillus velezensis* A4 increased by 67.8% after adding PEG4000 (polyethylene glycol) to the medium. However, little attention has been given to the regulatory mechanisms underlying cellulase biosynthesis in PEG-exposed microbial cells.

As a secondary messenger, Ca^2+^ is an important intracellular signaling molecule in microorganisms (Thewes 2014). Under the condition of added organic solvents, intracellular Ca^2+^ levels in *T. reesei* were involved in cellulase biosynthesis (Chen et al. 2018; Stranks 1973). Ca^2+^ is the key component of the calcium signal transduction pathway (Sasanuma and Suzuki 2016). Chen et al. (2016) demonstrated that increased cytosolic Ca^2+^ levels could activate *crz1* (a calcineurin-responsive zinc finger 1-encoding gene related to the calcium signaling pathway), then the activated Crz1 enters the nucleus and binds directly to the upstream regions of the cellulase gene *cbh1*. Several studies have shown that the calcineurin-Crz1 signaling cascade is ubiquitous in eukaryotes and can be activated by different external stimuli, such as Ca^2+^ (Soriani et al. 2008), Mn^2+^ (Cramer et al. 2008), caffeine (Calvo et al. 2009), and antifungal drugs (Edlind et al. 2002). However, the mechanism by which the calcium signaling pathway regulates cellulase biosynthesis in microbial cells induced by PEG8000 remains unclear.

In this study, the effect of PEG8000 on cellulase production and the underlying regulatory mechanism of *T. reesei* CICC2626 were investigated, the response of calcium signaling to PEG8000 was analyzed, and a potential mechanism by which PEG8000 induces cellulase production through calcium signal transduction in *T. reesei* was proposed. These results provide novel and valuable insights into effective methods to enhance cellulase production.

## Materials and methods

### Strains and growth conditions

*Escherichia coli* DH5α purchased from TransGen Biotech, Beijing, China, was used for plasmid amplification. *T. reesei* CICC2626 was purchased from China Industrial Microbial Species Conservation and Management Center (CICC). Luria–Broth (LB) medium and YPD medium (yeast extract 10 g/L; peptone 20 g/L; glucose 20 g/L) were adopted to cultivate *E. coli* DH5α and *T. reesei*, respectively. Minimal medium (MM) (KH_2_PO_4_ 6 g/L; (NH_4_)_2_SO_4_ 3 g/L; MgSO_4_·7H_2_O 1 g/L; CaCl_2_ 1 g/L; ZnSO_4_·7H_2_O 0.01 g/L, MnSO_4_·6H_2_O 0.01 g/L, CuSO_4_·7H_2_O 0.01 g/L, pH 6.0) with 2% glucose was used to assess the effect of PEG8000 on hyphal growth. The reagents used above are purchased from Sangon Biotech Co. Ltd. (Shanghai, China).

Before the experiment, the seed solution of *T. reesei* was cultured in YPD medium at 30 °C and 150 rpm for 24 h. Then, mycelia of about 0.1 g were collected by centrifugation at 5000 × *g* and washed using 0.9% NaCl solution. The collected mycelia were inoculated to 100 mL MM medium containing 2% (w/v) Avicel (Sigma, Shanghai, China) as the sole carbon source supplemented with 0% (control), 0.5%, 1.5%, 2%, or 3% (w/v) PEG8000. The corresponding osmotic pressure was 203, 206, 229, 236, and 268 mOsm/kg was measured by FM-8P automatic freezing osmometer (Shanghai Medical Instruments Co., Shanghai, China). The culture solution was aerobically incubated at 150 rpm in a shaker with constant temperature of 30 °C. The samples were collected on the third, fourth, and fifth day after inoculation for detecting enzymatic activity, extracellular protein concentration, and biomass, respectively, or on the 36th and 48th hour for RT-qPCR analyses. To identify the effects of Ca^2+^ channel of plasma membrane and cytosolic Ca^2+^ level on cellulase biosynthesis of *T. reesei* CICC2626, LaCl_3_ (a plasma membrane Ca^2+^ channel blocker) or EGTA (ethylene glycol tetraacetic acid, a putative extracellular Ca^2+^ chelator) (Aladdin, Shanghai, China) as an Ca^2+^ inhibitor at a concentration of 10 mM was individually added into liquid MM supplemented with 1.5% (w/v) PEG8000 after culture of 1 day, then the culture solution was cultivated for 5 days.

### Determination of fungal growth, enzymatic activity, protein concentration, and biomass

Five milliliters of sterile water was added into the YPD plate of *T. reesei* being cultured for 3 days, and the mycelia and spores growing on the surface of the plate were gently scraped off from the plate surface. The suspension containing mycelia and spores was filtered through four layers of sterilized gauze, and the filtrate containing spores was collected. The spore count was determined using a blood cell counting plate, and the spore suspension was diluted with sterile water to obtain a concentration of 1 × 10^6^ CFU (colony-forming units)/mL. Next, 5 μL (5 × 10^3^ CFU) of diluent was inoculated onto MM plates and further incubated at 30 °C for 4 days. The radius of each colony was measured using Vernier calipers.

One milliliters of the above-culture solutions supplemented with different amounts of PEG8000 was taken out and filtered through a 0.22-μm microfiltration membrane, and the filtrate as crude enzyme solution was then collected for the following test. The enzymatic activity and extracellular protein concentration in the filtrate were determined. Total cellulase (filter paper-hydrolyzing activity [FPase]) and endoglucanase (carboxymethyl cellulase activity [CMCase]) levels were determined based on the reducing sugars produced using Whatman filter paper and CMC-Na as the substrate. To test FPase activity, Whatman filter paper (1 × 5 cm) and 0.2 mL of the filter liquor were mixed in citrate buffer (pH 4.8), and incubated at 50 °C for 1 h. To detect CMCase activity, 1 mL of 2% (w/v) CMC-Na (Cowin Biotech Co. Ltd., Beijing, China) and 0.2 mL of filter liquor were mixed and incubated at 50 °C for 30 min. The amount of released reducing sugars was determined using the dinitrosalicylic acid (DNS) method (Miller 1959). β-Glucosidase activity was assayed using *p*-nitrophenyl β-d-glucopyranoside (*p*-NPG) (Aladdin, Shanghai, China) as described previously (Rajasree et al. 2013). For xylanase determination, 2 mL of 1% (w/v) xylan (Sangon Biotech Co. Ltd., Shanghai, China) and 0.5 mL crude enzyme solution were incubated at 40℃ for 1 h (Bailey et al. 1992). One unit of enzyme activity was defined as the enzyme quantity required to produce 1 μmoL glucose, *p*NP(*p*-nitrophenol), or xylose equivalents in 1 min. According to the manufacturer’s instructions, protein concentrations were determined using a total protein quantitative assay kit (Sangon Biotech, Shanghai, China).

To measure biomass, the Eppendorf tube (size 5 mL) was first dried at 40 °C in a vacuum oven till a constant weight (*w*_*1*_), and then 4 mL MM medium culture solution was centrifuged at 10,000 × *g* at 4 °C for 10 min in a dried Eppendorf tube to collect mycelia. Mycelia were washed three times with sterile water and dried at 80 °C in a vacuum oven until a constant weight (*w*_2_) was reached. The biomass in the culture solution was calculated using the following formula

1 $$w={w}_{2}-{w}_{1}$$

### Synergistic hydrolysis of alkali-pretreated rice straw

The rice straw was dried at 50 °C for 24 h, crushed in a mixer, and passed through a 20-mesh sieve. The powder of rice straw was pretreated with NaOH solution (10%, w/v) at a solid/liquid ratio of 1:15 at 80 °C for 2 h. Then, the mixture was filtered with four layers of gauze to collect the solids and rinsed with distilled water until its pH became neutral. After drying at 50 °C, the solids were used for the subsequent experiments. Composition of the rice straw was determined according to the standardized method of the National Renewable Energy Laboratory (NREL, Golden, CO, USA) (Xu et al. 2017). The composition analysis of the raw rice straw showed that the content of cellulose, hemicellulose, and lignin was 37.8, 35.2, and 15.4%, respectively. After alkaline pretreatment, it contained 62.5% cellulose, 23.4% hemicellulose, and 7.8% lignin. The yield of treated residue from rice straw was 76.1%.

Synergistic hydrolysis of alkali-pretreated rice straw by commercial cellulase from *A. niger* (Aladdin, Shanghai, China) and the crude enzyme (*T. reesei* CICC2626 cultivated solution from 0 or 1.5% PEG8000 supplement was filtered by 0.22-μm membrane) was performed in 50 mM citrate phosphate buffer (pH 4.8) in a total volume of 20 mL. During hydrolysis, the initial biomass concentration of alkali-pretreated rice straw was 15% (w/v), the enzyme loads for commercial cellulase and crude enzyme were both 15 FPU/g (filter paper activity unit /g) substrate, and the mixture was incubated at 50 °C with 200 rpm agitation for 48 h. Furthermore, commercial cellulase (30 FPU/g substrate) and crude enzyme (30 FPU/g substrate) were incubated individually with 15% (w/v) alkali-pretreated rice straw, and the reactions were performed under the same conditions. Samples were withdrawn at regular intervals, and the supernatant was collected for the reducing sugar assay.

### RNA isolation and RT-qPCR

The transcriptional level of mRNA was detected through RT-qPCR. The total RNA was extracted by Trizol reagent (TaKaRa, Dalian, China). cDNA synthesis of the total RNA was performed using the PrimeScript™ RT reagent Kit with gDNA Eraser Kit (TaKaRa Dalian, China) as per the manufacturer’s instructions. RT-qPCR was conducted using an ABI StepOne thermocycler (Applied Biosystems, Foster City, CA, USA), gene transcription was then analyzed using SYBR green assays. The *actin* (ID: 18482341) gene was applied as internal references to normalize the gene transcription of targets genes. The sequences of the primers for RT-qPCR are shown in Supplementary file 2: Table S1. The relative gene expression levels were analyzed using the 2^−ΔΔCt^ method (Livak 2001).

### Transcriptome analysis

The cells were harvested by centrifugation from cultures grown for 36 h in liquid MM medium containing 1% Avicel as the sole carbon source with the addition of 0 (control) or 1.5% PEG8000. Total RNA was respectively extracted from the control group (repeated samples, CK1, CK2, and CK3) and the PEG8000 group (repeated samples, T1, T2, and T3). Then, the extracted RNA was sequenced by Personal Biotechnology Co., Ltd. (Shanghai, China). The sequence reads were mapped to the *T. reesei* reference genome (https://www.ncbi.nlm.nih.gov/genome/?term=Trichoderma%20reesei) for bioinformatic analysis. The raw whole transcriptome shotgun sequencing data (BioProject ID PRJNA761525) were deposited in the NCBI Sequence Read Archive (SRA) database. The differential analysis of gene expression was performed by DESeq software (version 1.18.0) (Zhang et al. 2014), and the differentially expressed genes were screened out according to expression fold difference log_2_ |fold change|> 1 and significance *p*-value < 0.05 (Robinson et al. 2010). To confirm RNA-seq data, five upregulated genes and five downregulated genes were randomly selected using the RNA extracted above as the template for RT-qPCR analysis. These genes and primer sequences used for verifying transcriptome data are shown in Supplementary file 2: Table S1. The information of selected genes and their differential expression levels are shown in Supplementary file 2: Table S2. All experiments were conducted in three biological and technical replications.

### Construction of plasmids and transformation

To construct a *crz1* (ID:18,484,666) deletion mutant, the upstream (− 1 to − 707 bp) and downstream (+ 2149 to + 2863 bp) fragments of *crz1* from the genome of *T. reesei* CICC2626 were amplified using PrimeSTAR® Max DNA Polymerase (TaKaRa, Dalian, China). First, the upstream and downstream fragments were ligated into hygromycin cassette using overlap extension PCR method to form a fusion gene fragment (U*crz*1-*hyg*-D*crz*1). Subsequently, the fusion gene fragment was inserted into plasmid *pEASY*-Blunt Zero Cloning Vector (TransGen Biotech, Shanghai, China) to obtain the gene deletion cassette. The knockout of *crz1* in *T. reesei* was performed using the *Agrobacterium*-mediated transformation method as described previously (Wang et al. 2020) (Supplementary file 1: Fig. S1a). Positive transformants on YPD plates were selected out using hygromycin B and ampicillin. Then, the putative *crz1* disruption mutants (Δ*crz1*) generated by double crossover were verified by diagnostic PCR. The used primers Crz1-CF1, Crz1-CR1, Crz1-CF2, and Crz1-CR2 are shown in Supplementary file 1: Fig. S1b.

### Determinations of cytosolic Ca^2+^ and ROS levels

The level of cytosolic Ca^2+^ in* T*. *reesei* was measured using a Ca^2+^ fluorescent probe (Fluo-3AM); the fluorescent intensity of the Fluo-3AM-labelled cells was detected by using a LS-55 spectrophoto fluorometer (PerkinElmer, Waltham, USA) with a 488-nm excitation wavelength and 525-nm emission wavelength. Intracellular Ca^2+^ green fluorescence was imaged using an inverted fluorescent microscope (Leica, Wetzlar, Germany). The endogenous reactive oxygen species (ROS) level was quantified by ROS Assay Kit as previously described (Cui et al. 2020).

### Statistical analysis

Mean values ± standard deviation of triplicate measurement data was shown in this work. Duncan’s multiple-range test was used for multiple comparisons. Student’s *t* test was used to measure the significance of the difference between the treatment means. *p* < 0.05(*) or* p* < 0.01(**) were considered significant.

## Results

### Cellulase activities, extracellular protein concentration, and biomass in T. reesei CICC2626 with the addition of PEG8000

**Fig. 1:**
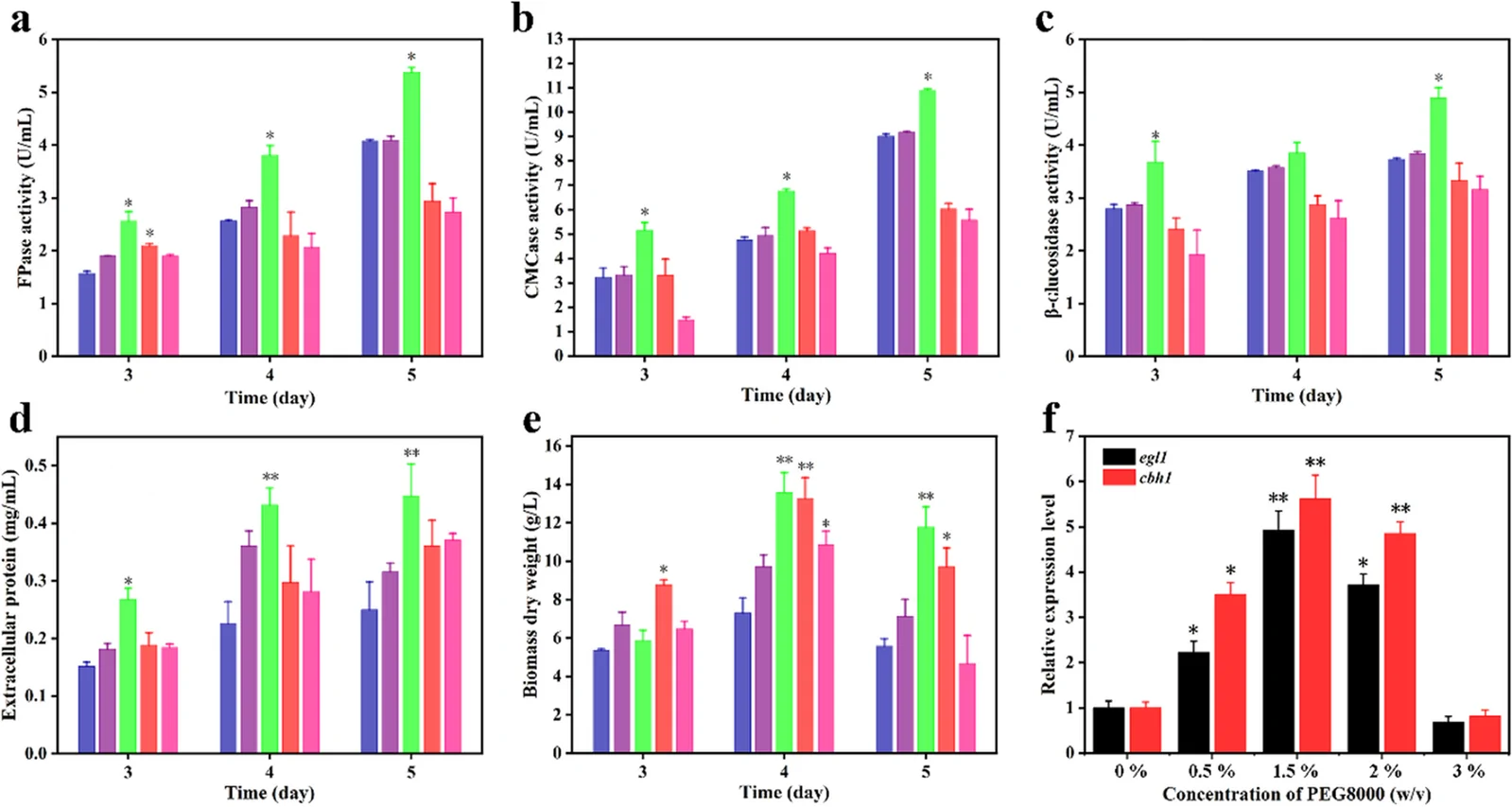
Effects of different concentrations of PEG8000 at the final concentrations of 0, 0.5, 1.5, 2, and 3% (w/v) on the FPase activity (**a**), CMCase activity (**b**), β-glucosidase activity (**c**), extracellular protein concentration (**d**), biomass (**e**), and the transcription levels of cellulase genes (**f**) of* T*. *reesei* CICC2626. The seed solution of *T*. *reesei* CICC2626 was cultured in YPD medium for 24 h, transformed to fresh MM medium containing 2% (w/v) Avicel with 0 to 3% PEG8000, and then cultivated for 36 to 120 h. Blue bar, addition of 0% (w/v) PEG8000; purple bar, addition of 0.5% (w/v) PEG8000; green bar, addition of 1.5% (w/v) PEG8000; red bar, addition of 2% (w/v) PEG8000; pink bar, addition of 3% (w/v) PEG8000. Values are the mean ± SD of the results from three independent experiment. Asterisks indicate significant differences (*p < 0.05, **p < 0.01, Student’s *t* test)

To evaluate the effect of PEG8000 on cellulase production by *T*. *reesei*, the activities of FPase, CMCase, and β-glucosidase in the MM medium were measured (Fig. 1a–c). It can be found that cellulase activities were gradually increased with an increase in PEG8000 concentration from 0 to 1.5% (corresponding to osmotic pressure 203, 206, 229 mOsm/kg) at all times. In particular, the activities of FPase (5.4 ± 0.4 U/mL), CMCase (10.9 ± 1.2 U/mL), and β-glucosidase (4.9 ± 0.5 U/mL) supplemented with 1.5% PEG8000 were 1.3-, 1.2-, and 1.3-fold those of the control group, respectively. Those indexes in the control group were 4.1 ± 0.3, 9.0 ± 0.8, and 3.7 ± 0.3 U/mL after incubation for 5 days. Because cellulases are extracellular enzymes, the extracellular protein concentration can be used to monitor cellulase production by microbial cells (Mishra et al. 2021). As shown in Fig. 1d, the changes in extracellular protein concentration were consistent with those of cellulase activity. The protein concentration reached a maximum of 0.5 ± 0.1 mg/mL with 1.5% PEG8000 treatment after incubation for 5 days, which was 1.7-fold that of the control (0.3 ± 0.1 mg/mL). However, the protein concentration significantly decreased with a further increase in PEG8000 concentration. The results indicated that cellulase production by *T. reesei* drastically increased with a final concentration of 1.5% PEG8000 supplementation in the MM medium.

The most abundantly secreted cellulases in *T. reesei* are cellobiohydrolases (EC 3.2.1.91) and endo-β-1,4-endoglucanases (EC 3.2.1.4) (Häkkinen et al. 2012). To clarify whether the improved cellulase activity could be attributed to the biomass or expression levels of the cellulase genes, the biomass and expression levels of the two main cellulase genes (*egl1* encoding endoglucanase I, *cbh1* encoding cellobiohydrolase I) were determined. As shown in Fig. 1e, the biomass of *T. reesei* gradually increased with a final concentration of 0–1.5% (w/v) PEG8000 supplementation in the MM medium. The maximal biomass (13.6 ± 1.2 g/L) was achieved after incubation for 4 days when 1.5% PEG8000 was added to the culture medium, which was 1.9-fold that of the control group (7.3 ± 0.9 g/L). PEG has good biocompatibility and biodegradability. In the environment, PEG does not cause pollution to soil, water, and air because it is broken down into smaller molecules by microorganisms. In the biological body, PEG can also be hydrolyzed by enzymes into harmless substances such as water and carbon dioxide, which will not cause harm to the human body (Gao et al. 2022). Some studies have shown that methanogenic bacteria can degrade PEG (Dwyer et al. 1983). Therefore, we hypothesized that PEG8000 may be decomposed in the growth process of *T. reesei*, thus increasing the biomass. The mycelial morphology of *T*. *reesei* grown on YPD plates containing PEG8000 is shown in Supplementary file 1: Fig. S2. There was little difference in colony radii among the 0, 0.5, and 2% groups. However, when 3% PEG8000 was added, the colony radius decreased. The maximal colony radius (3.6 ± 0.1 cm) was achieved after incubation for 4 days supplemented with 1.5% PEG8000, which was 1.1-fold that of the control group (3.2 ± 0.1 cm). In addition, the hyphal morphology of 0.5, 1.5, and 2% PEG8000 was fuller and stronger than that of 0 and 3% PEG8000, and the mycelial growth state was the worst when supplemented with 3% PEG8000. This may be owing to the high osmotic pressure caused by adding 3% PEG8000 (268 mOsm/kg), which inhibits mycelial growth. As illustrated in Fig. 1f, the transcript levels of two cellulase genes (*egl1* and *cbh1*) displayed a sharp upregulation. In the 1.5% PEG8000 group, the transcription levels of the two genes were 4.9- and 5.6-fold higher than those in the control group. However, the transcriptional levels in cells supplemented with 3% PEG8000 were only 0.7- and 0.9-fold higher than in control. The endogenous ROS content was significantly reduced with an increase in PEG8000 concentration from 0 to 1.5%. However, ROS levels in the 3% PEG8000 treatment were observably higher than those in the 0% (Supplementary file 1: Fig. S3). These results suggest that higher PEG8000 levels may increase intracellular ROS levels, inhibiting mycelial growth and cellulase production. Based on these results, the increase in cellulase activity in *T. reesei* induced by PEG8000 can be ascribed to an increase in biomass and the overexpression of cellulase genes. The optimal final concentration of PEG8000 for enhancing cellulase production was 1.5%.

### Synergistic effect with commercial cellulase in the hydrolysis of alkali-pretreated rice straw

**Fig. 2:**
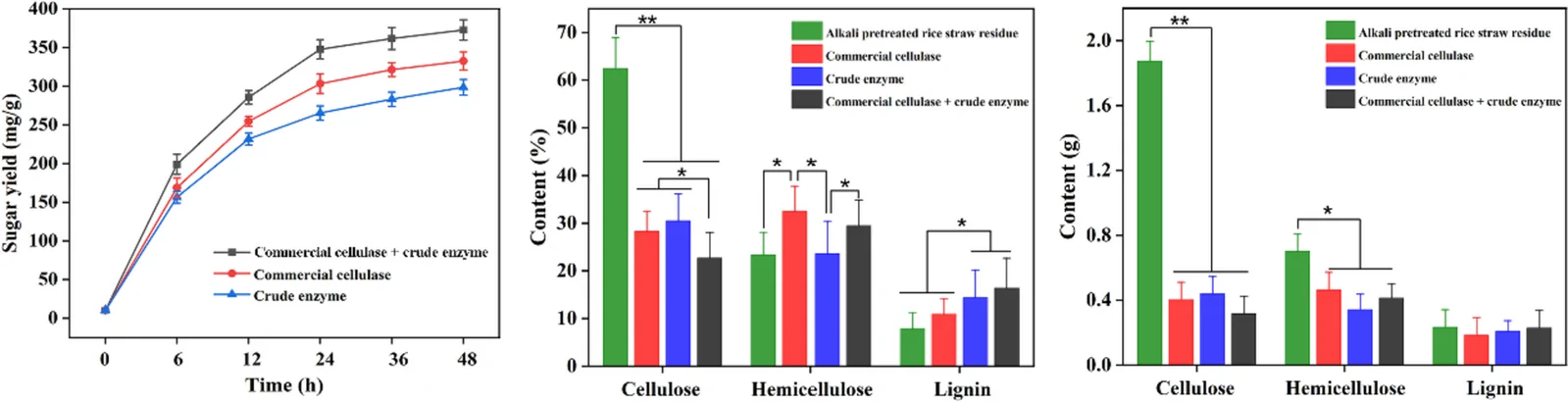
Synergistic hydrolysis of pretreated rice straw by crude enzyme and commercial cellulase from 1.5% PEG8000 supplement culture. **a** The concentration of reducing sugar after hydrolysis. **b**–**c** The percentage and weight of cellulose, hemicellulose, and lignin in alkali-pretreated rice straw residues after hydrolysis. The hydrolysis reaction was carried out with 15% (w/v) alkali-pretreated rice straw in a 20-mL system. After the hydrolysis reaction, the treated residue yield of groups commercial cellulase, crude enzyme, and commercial cellulase + crude enzyme from rice straw was 1.43, 1.45, and 1.40 g respectively. Values are the mean ± SD of the results from three independent experiments. Asterisks indicate significant differences (*p < 0.05, Student’s *t* test)

Rice straw is an abundant agricultural residue and is a promising feedstock for the production of biofuels and other chemicals due to its high cellulose and hemicellulose content. In general, the hydrolysis efficiency of rice straw can be enforced through the synergistic effect of the collected crude enzyme and the commercial cellulase (Liu et al. 2022). Here, the synergistic effect of the crude enzyme (*T*. *reesei* CICC2626 cultivated solution from 1.5% PEG8000 supplement was filtered by 0.22-μm membrane) and commercial cellulase on the degradation of alkali-pretreated rice straw was evaluated. After hydrolysis for 48 h, the reducing sugar concentration for synergy of commercial cellulase (15 FPU/g substrate) and 15 FPU/g substrate of crude enzyme was 372.7 mg/g, which was 1.1- and 1.2-fold those of commercial cellulase (332.5 mg/g) and crude enzyme (298.6 mg/g), respectively (Fig. 2a). This is because the rice straw after alkali treatment contains a large amount of hemicellulose (23.4%), which can be hydrolyzed by xylanase in crude enzyme (Supplementary file 1: Fig. S4), resulting in the exposed cellulase being easier to hydrolyze into reducing sugar by cellulase.

In addition, the contents of cellulose, hemicellulose, and lignin in alkali-pretreated rice straw residue were analyzed before and after hydrolysis. As shown in Fig. 2b and c, the content of cellulose significantly decreased after enzymatic hydrolysis, and the minimal content of cellulose (22.7%, 0.32 g) was obtained in the treatment with commercial cellulase and crude enzyme, which was significantly lower than the initial content (62.5%, 1.88 g). The obtained result shows that the mixture enzymes of commercial cellulase and crude enzyme could improve the hydrolysis efficiency of cellulose. Therefore, cellulases produced by *T*. *reesei* CICC2626 could collaborate with commercial cellulase in the biotransformation of lignocellulosic biomass. Of note, the hemicellulose content in the residues after hydrolysis by crude enzyme (23.6%, 0.34 g) was significantly lower than that of commercial enzyme (32.5%, 0.46 g); however, the cellulose content of residues hydrolyzed by crude enzyme (30.5%, 0.44 g) was slightly higher than that by commercial cellulase (28.3%, 0.40 g). This can be attributed to xylanase produced by* T*. *reesei* CICC2626 (Supplementary file 1: Fig. S4), which promoted the hydrolysis of hemicellulose. In addition, the synergistic effect of the crude enzyme (*T. reesei* CICC2626 cultivated solution from 0% PEG8000 supplement was filtered by 0.22-μm membrane) and commercial cellulase on the degradation of alkali-pretreated rice straw was also evaluated. As shown in Supplementary file 1: Fig. S5, the change trend of reducing sugar concentration and the percentage or weight of cellulose, hemicellulose, and lignin in alkali-pretreated rice straw residues after hydrolysis was consistent with that of 1.5% PEG8000 supplement culture. These results indicate that crude enzyme and commercial cellulase possessed clear synergistic effects on alkali-pretreated rice straw hydrolysis.

### Transcriptome of T. reesei CICC2626 supplemented with PEG8000

To gain insight into the potential mechanism by which the expression of cellulase genes is regulated by PEG8000 addition, two transcriptomes of *T. reesei* CICC2626 cultured in media without PEG8000 as control and with 1.5% PEG8000 were compared. The transcriptome data indicated that 3850 genes showed differential expression between the two groups with a log_2_ |fold change|> 1 and a significant *p*-value < 0.05. Of the 3850 differentially expressed genes (DEGs), 2081 were upregulated and 1769 were downregulated (Supplementary file 1: Fig. S6 and Supplementary file 2: Table S2). Furthermore, functional categorization and metabolic processes of the 3850 DEGs were performed using Gene Ontology terms (https://urldefense.com/v3/__https://geneontology.org/__;!!NLFGqXoFfo8MMQ!pW8xTxVrhoJQuCYrJLGO5j6gT_5ysXjbSrX6ZF01JwmkI2Qstmc2AkywLpdYvxPvxxB8cb9C1zYR05Kwe8tpV_t8_nTf$) and KEGG (https://urldefense.com/v3/__https://www.genome.jp/kegg/__;!!NLFGqXoFfo8MMQ!pW8xTxVrhoJQuCYrJLGO5j6gT_5ysXjbSrX6ZF01JwmkI2Qstmc2AkywLpdYvxPvxxB8cb9C1zYR05Kwe8tpV6HHoSwX$) pathway enrichment analysis, respectively. Analysis using Gene Ontology (GO) terms showed that in the presence of PEG8000, most of the DEGs were related to ribosome, structural constituent of ribosome, and small molecule metabolic process (Supplementary file 2: Table S3). On the other hand, KEGG pathway enrichment analysis showed that most of the DEGs were related to pentose phosphate pathway, MAPK signaling pathway, calcium signaling pathway, and mRNA surveillance pathway with 1.5% PEG8000 treatment (Supplementary file 2: Table S4). Five upregulated and five downregulated genes were selected for RT-qPCR analysis to verify the RNA-seq data (Supplementary file 1: Fig. S7). The validated DEGs were consistent with the RNA-seq results, supporting the validity of the RNA-seq data.

In total, 12 lignocellulose degradation–related genes were significantly upregulated in the 1.5% PEG8000 addition group (Table 1). Three main cellobiohydrolase-encoding genes (*cel7a*, *cel6a* and *cbh*; IDs: 18483782, 18488147, and 18483234), six endoglucanase-encoding genes (IDs: 18483009, 18488225, 18483598, 18482842, 18483318, and 18484770; including *cel61b*, *cel61a*, *cel12a*, *cel5a*, *cel7b*, and *egl*), two β-glucosidase-encoding genes (IDs: 18483230 and18482947; including *cel3b* and *cel1a*), and an α-xylosidase gene (ID: 18487910) were upregulated with 1.5% PEG8000 addition. However, 2 endoglucanase-encoding genes (IDs: 18485669 and 18489491; including *cel5b* and *egl2*) and a β-xylosidase gene (ID: 18484697) were downregulated in the 1.5% PEG8000 addition group. In addition, there was no significant difference in the expression of *cel6b* (ID: 18484386; encoding endoglucanase) between the two groups. The transcriptome data further identified cellulase activities and RT-qPCR result (Fig. 1). It reveals that the presence of 1.5% PEG8000 significantly upregulated the transcription levels of the lignocellulose degradation–related genes.

**Table 1:** Log2-fold changes in major lignocellulose degradation–related genes transcripts induced by PEG8000

Gene ID	Annotation	Log_2_ fold change (CK^a^/T^b^)	Regulation
18,483,782	CellobiohydrolaseI CBH1/*cel7a*	5.51	Up
18,488,147	CellobiohydrolaseII CBH2/*cel6a*	2.88	Up
18,483,234	Cellobiohydrolase/*cbh*	6.20	Up
18,483,009	Endoglucanase/*cel61b*	3.78	Up
18,488,225	Endoglucanase/*cel61a*	4.49	Up
18,483,598	Endoglucanase/*cel12a*	5.02	Up
18,482,842	Endoglucanase/*cel5a*	2.19	Up
18,483,318	Endoglucanase/*cel7b*	2.03	Up
18,484,770	Endoglucanase/*egl*	1.98	Up
18,483,230	β-Glucosidase/*cel3b*	4.34	Up
18,482,947	β-Glucosidase/*cel1a*	2.47	Up
18,487,910	α-Xylosidase	7.11	Up
18,485,669	Endoglucanase/*cel5b*	− 1.96	Down
18,489,491	Endoglucanase VIII/*egl2*	− 2.96	Down
18,484,697	β-Xylosidase	− 1.45	Down
18,484,386	Endoglucanase/*cel6b*	− 0.46	—

^a^*CK*, gene expression level in control group; ^b^*T*, gene expression level in PEG8000 group

Notably, all four genes in the calcium signaling pathway were upregulated by adding of PEG8000 compared to those in the control (Supplementary file 2: Table S2). Therefore, we investigated the relationship between the upregulated expression of cellulase genes and calcium signaling in the presence of PEG8000. As shown in Table 2, the gene *plc-e* (ID: 18484061), which encodes a phospholipase C protein that generates inositol-1,4,5-trisphosphate (IP3) to regulate calcium release from intracellular pools (Schmoll 2008), was remarkably upregulated in the 1.5% PEG8000 group compared to that in the control. In addition, three genes *cam* (ID: 18489174; encoding calmodulin), *can* (ID: 18486548; encoding calcineurin), and *crz1* (ID: 18484666; encoding transcription factor Crz1 which are related to calcium signaling pathway) were upregulated in the 1.5% PEG8000 group.

**Table 2:** Log2-fold changes in calcium signaling pathway–related genes transcripts induced by PEG8000

Gene ID	Annotation	Log_2_ fold change (CK^a^/T^b^)	Regulation
18,484,061	Phospholipase C/*plc-e*	3.29	Up
18,489,174	Calmodulin /*cam*	2.78	Up
18,486,548	Calcineurin /*can*	2.19	Up
18,484,666	Transcription factor Crz1 /*crz1*	2.58	Up

^a^*CK*, gene expression level in control group; ^b^*T*, gene expression level in PEG8000 group

The transcription levels of *plc-e*, *cam*, *can*, and *crz1* were analyzed using RT-RT-qPCR (Supplementary file 1: Fig. S8). The RT-qPCR results were consistent with the whole-transcriptome shotgun sequencing analysis; this suggests that 1.5% PEG8000 treatment upregulated the intracellular cytosolic Ca^2+^ level in *T. reesei* CICC2626, and that calcium signaling transduction pathway might have been involved in expression regulation of these cellulase genes.

### Effect of cytosolic Ca^2+^ level on cellulase production by T. reesei CICC2626 with PEG8000 treatment

Based on the transcriptional profiling results, it can be deduced that PEG8000 influences cytosolic Ca^2+^ levels. The cytosolic Ca^2+^ levels were measured using the Fluo-3 AM fluorescent dye method to verify this (Wang et al. 2020). To determine whether the cytosolic Ca^2+^ burst was induced by PEG8000-mediated cellulase biosynthesis, EGTA, a putative extracellular Ca^2+^ chelator, or LaCl_3_, a Ca^2+^ channel blocker of the plasma membrane, was added to the culture medium.

**Fig. 3:**
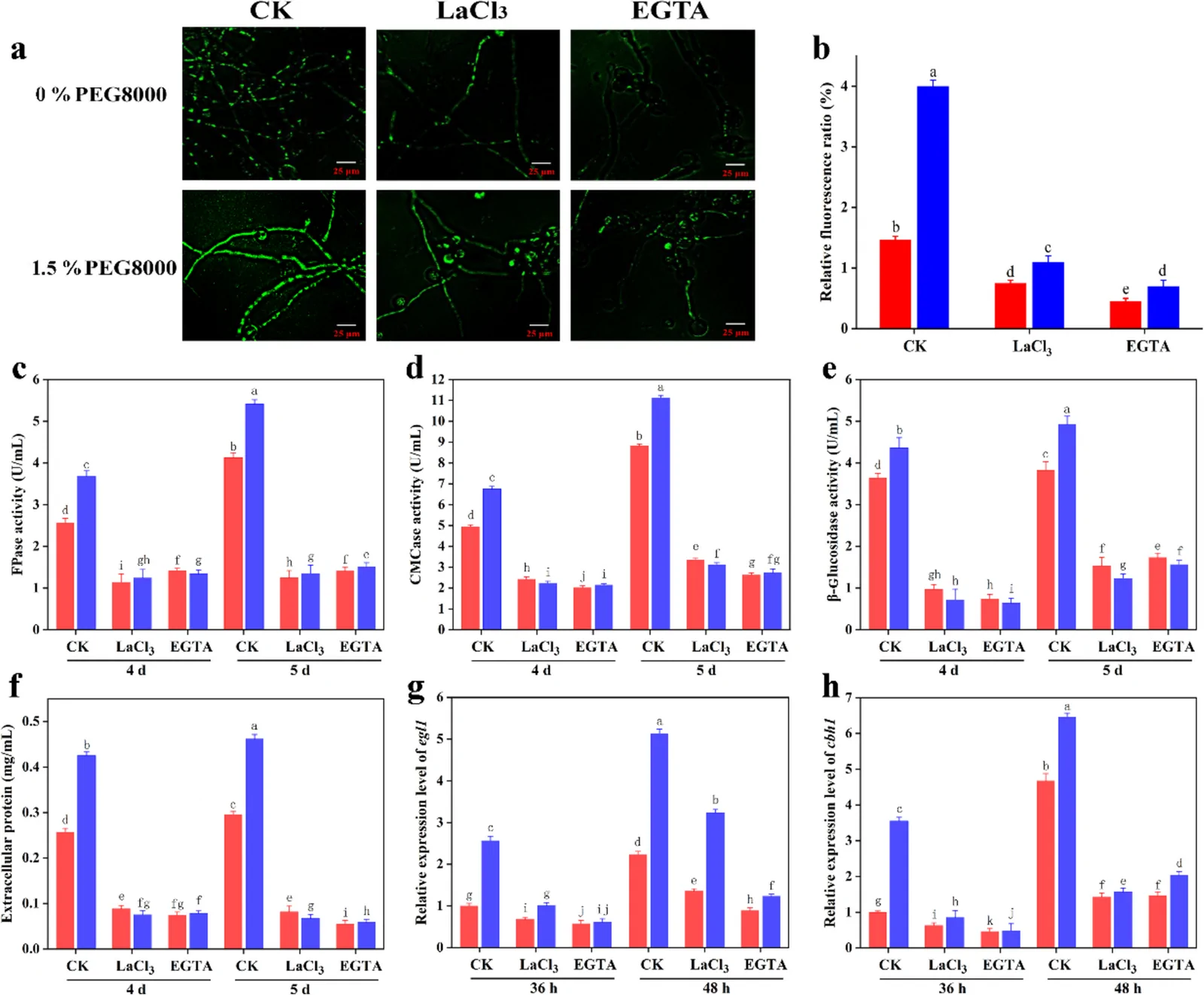
Effects of Ca^2+^ inhibitors on the fluorescence intensity (**a**), relative fluorescent ratio (**b**), FPase activity (**c**), CMCase activity (**d**), β-glucosidase activity (**e**), extracellular protein concentration (**f**), and expression levels of *egl1* (**g**) and *cbh1* (**h**) of *T*. *reesei* CICC2626. *T*. *reesei* CICC2626 was cultured in YPD medium for 24 h, transformed to fresh MM containing 2% (w/v) Avicel with 0 or 1.5% (w/v) PEG8000 and 0 or 10 mM LaCl_3_/EGTA, and cultivated for 36 to 120 h. For detection, Fluo-3 AM was used, and fluorescence intensity was detected using inverted fluorescence microscopy. Blue bar, addition of 1.5% (w/v) PEG8000 in strain; red bar, addition of 0% (w/v) PEG8000 in strain. CK, 0% (w/v) PEG8000 (control) and 1.5% (w/v) PEG8000 group. Green fluorescence represents free cytosolic Ca^2+^. Values are mean ± standard deviation (*n* = 3). Different letters indicate significant differences between the columns (*p* < 0.05, Duncan’s multiple-range test)

As shown in Fig. 3a and b, the green fluorescence intensity of hyphae treated with 1.5% PEG8000 was stronger than that of the control (without PEG8000). However, the green fluorescence intensity of mycelia treated with PEG8000 + LaCl_3_ or PEG8000 + EGTA was markedly decreased compared to that of mycelia exposed only to PEG8000. This result demonstrates that the cytosolic Ca^2+^ burst induced by PEG8000 was effectively blocked by adding LaCl_3_ or EGTA; this revealed that PEG8000 increased cytosolic Ca^2+^ levels in *T. reesei* CICC2626 cells.

As shown in Fig. 3, the cellulase activity, extracellular protein concentration, and transcriptional levels of *egl1* and *cbh1* in the PEG8000 + LaCl_3_ and PEG8000 + EGTA groups were decreased compared with those in the CK groups (PEG8000 group, control group) at the same cultivation time. Specifically, the activities of FPase, CMCase, and β-glucosidase, and protein the concentration in the PEG8000 + LaCl_3_ and PEG8000 + EGTA groups were significantly decreased by 73.74–87.01% on the fifth day, respectively, compared to the CK groups (only PEG8000) (Fig. 3c–f). The transcriptional levels of *egl1* and *cbh1* in the PEG8000 + LaCl_3_ or PEG8000 + EGTA groups decreased by 75.72–75.93% than those of the PEG8000 group of the CK on the 48th hour of inoculation (Fig. 3g, h). However, the biomasses of the PEG8000 + LaCl_3_ and PEG8000 + EGTA groups were not significantly different from that of the CK groups (PEG8000 group, control group) at the same cultivation time (Supplementary file 1: Fig. S9). These data indicated that the decrease in cellulase activity and transcriptional levels of cellulase genes was independent of biomass after adding LaCl_3_ and EGTA. These results demonstrated that blocking the cytosolic Ca^2+^ burst using Ca^2+^ inhibitors can significantly attenuate cellulase production in *T. reesei* exposed to PEG8000.

### Expression of cellulase gene mediated by calcium signaling with PEG8000 treatment

**Fig. 4:**
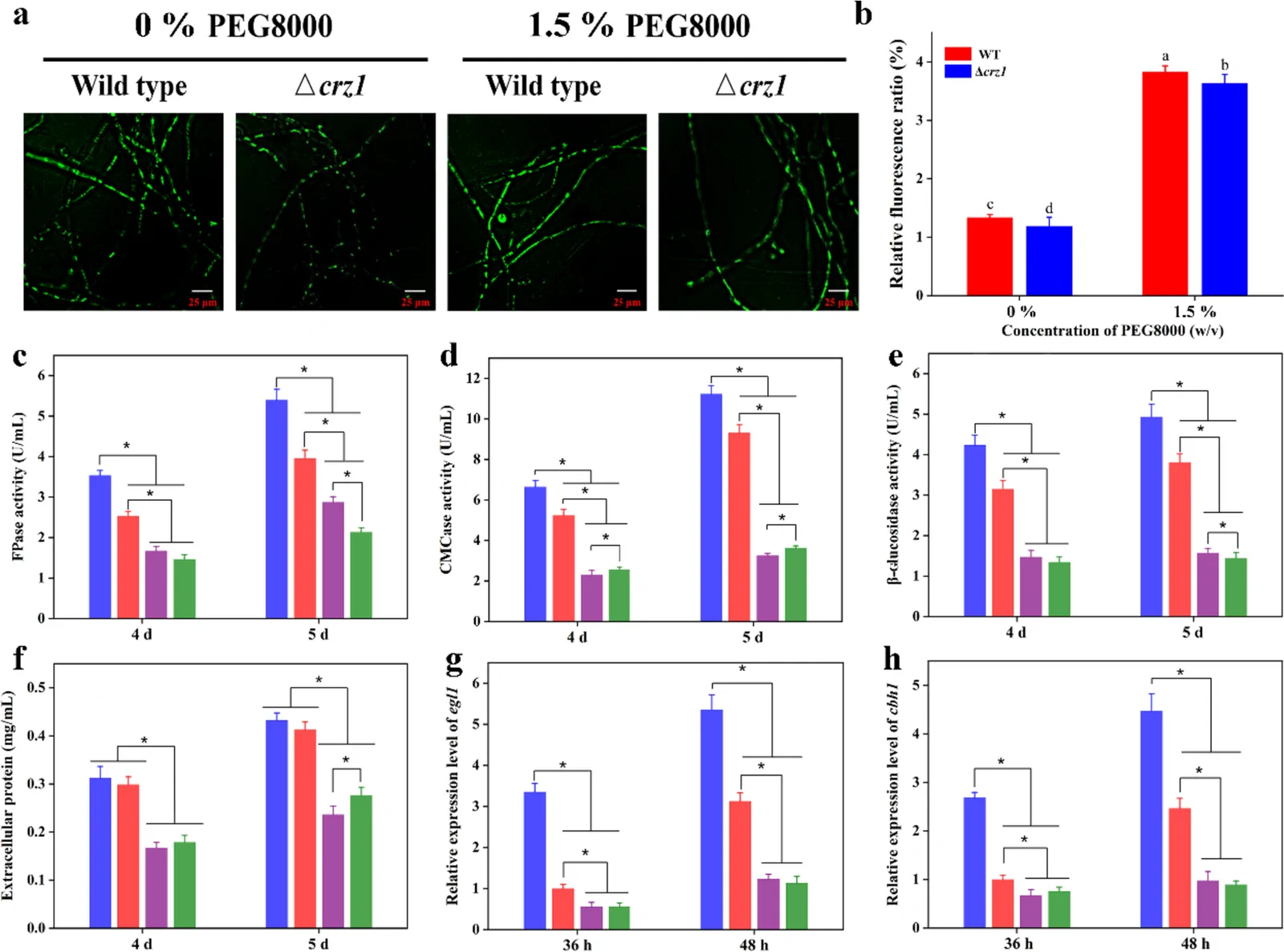
Effect of *crz1* on the fluorescence intensity (**a**), relative fluorescent ratio (**b**), FPase activity (**c**), CMCase activity (**d**), β-glucosidase activity (**e**), extracellular protein concentration (**f**), and expression levels of *egl1* (**g**) and *cbh1* (**h**) of wild type and Δ*crz1* strains. Wild-type and Δ*crz1* strains were cultured in YPD medium for 24 h, transformed to fresh MM containing 2% (w/v) Avicel with 0% or 1.5% (w/v) PEG8000, and cultivated for 36 to 120 h. Blue bar, addition of 1.5% (w/v) PEG8000 in wild-type strain; red bar, addition of 0% (w/v) PEG8000 in wild-type strain; purple bar, addition of 1.5% (w/v) PEG8000 in Δ*crz1*; green bar, addition of 0% (w/v) PEG8000 in Δ*crz1*. Green fluorescence represents free cytosolic Ca^2+^. Values are mean ± standard deviation (*n* = 3). Asterisks indicate significant differences (*p < 0.05, Student’s *t* test)

To further clarify the role of calcium signaling in cellulase synthesis in the presence of PEG8000, a *crz1* deletion mutant Δ*crz1* of *T. reesei* was constructed. As shown in Fig. 4a and b, the green fluorescence intensities of the wild type (WT) and Δ*crz1* strains exposed to PEG8000 were higher than those of the control (WT and Δ*crz1* strain without PEG8000), which further confirmed that PEG8000 induced an increase in cytosolic Ca^2+^ levels. Meanwhile, the intracellular fluorescence intensities of the WT and Δ*crz1* strains were almost the same in the control group and the PEG8000 groups, suggesting that PEG8000 induced an increase in the cytosolic Ca^2+^ level.

As illustrated in Fig. 4c–h, cellulase activities, protein concentration, and transcriptional levels of *egl1* and *cbh1* in Δ*crz1* + PEG8000 group or Δ*crz1* group were decreased, compared to those in WT + PEG8000 group or WT at all times. In the PEG8000 group, the activities of FPase, CMCase, β-glucosidase, and extracellular protein concentration in the WT strain were 1.6-, 1.7-, and 1.7-fold, respectively, for the Δ*crz1* strain on the 5th day of inoculation (Fig. 4c–f). Nevertheless, the biomass of the WT + PEG8000 or Δ*crz1* + PEG8000 groups was higher than that of the WT or Δ*crz1* strains without PEG8000 addition. The biomasses of the WT and Δ*crz1* strains were similar in the control group and PEG8000 group (Supplementary file 1: Fig. S10); this suggests that the cellulase activities of the Δ*crz1* strain treated with PEG8000 were remarkably decreased, and knockout *crz1* had little effect on strain growth. This may be due to the enhanced influence of other signaling pathways (such as MAPK signaling pathway) on the growth and development of cells after the deletion of *crz1* gene, and the activation of the enzyme coding genes related to the decomposition of PEG8000 to maintain the normal growth of cells. In addition, the transcription levels of *egl1* and *cbh1* of WT supplemented with PEG8000 were 4.1-fold and 4.6-fold than the indexes in Δ*crz1* strains treated with PEG8000 at 48 h (Fig. 4g, h). These data showed that the transcription levels of the cellulase genes were downregulated in Δ*crz1* strains compared to those in the WT strains treated with PEG8000. Taken together, these results demonstrated that the significant increase in levels of *egl1* and *cbh1* transcription induced by PEG8000, observed in the wild type, was abolished in the *crz1* deletion strain. Chen et al. (2018) reported that that calcium signaling plays a dominant role in cellulase overexpression in *T. reesei*. Based on the results of this and previous studies, it can be considered that the calcium signaling pathway is involved in cellulase biosynthesis in *T. reesei* treated with PEG8000.

## Discussion

High cellulase production by *T. reesei* requires inducers, and traditional inducers include cellulose and cellobiose (Derntl et al. 2017; Kubicek et al. 2009). PEG8000 can accelerate the elimination of amorphous cellulose and promote cellulase synthesis (Reese and Maguire 1969). Studies have shown that adding PEG can not only affect the biosynthesis of cellulase, but also reduce the adsorption capacity of cellulase to lignin so as to reduce the enzyme loading and promote the effective combination of enzyme and substrate (Luo et al. 2017). In addition, compared with organic solvent inducers such as *N,N*-dimethylformamide (DMF) (Chen et al. 2019), PEG has good biocompatibility and biodegradability (Gao et al. 2022), which ensures the safety of the biological process and the enzyme produced.

In this study, cellulase activities, extracellular protein concentration, biomass, and transcriptional levels of cellulase genes (*egl1* and *cbh1*) in *T*. *reesei* CICC2626 supplemented with 1.5% PEG8000 were significantly increased higher than those in the control group (Fig. 1); this implies that PEG8000 could be a novel inducer for *T. reesei* cellulase production. However, when PEG8000 was increased to 3% (268 mOsm/kg), enzyme activity, mycelial growth, and biomass of the cells decreased (Fig. 1), whereas intracellular ROS levels increased (Supplementary file 1: Fig. S3). Liu et al. (2022) confirmed that increased intracellular ROS levels were detrimental to cellulase production. It can be inferred that PEG8000 can promote growth and enzyme production under appropriate addition (0.5–1.5%), but high addition (3%) will cause osmotic stress, producing ROS, and affect mycelia’s growth and enzyme production.

Previous studies have shown that mixed enzymes containing cellulase and xylanase are more efficient than cellulase alone for decomposing lignocellulosic substrates (Long et al. 2018). Our research demonstrated that the crude enzyme produced by *T*. *reesei* CICC2626 contains xylanase activity (Supplementary file 1: Fig. S4). The results also indicated that crude enzyme mixed with commercial cellulase have the potential to be applied to the hydrolysis of other agricultural and forestry wastes (Fig. 2). Similar results were obtained for the crude enzymes produced by *Streptomyces* DSK59 (Budihal et al. 2015).

Transcriptome data revealed that 12 lignocellulose degradation–related genes were upregulated in the 1.5% PEG8000 addition group (Table 1). This result further suggested that the enhancement of cellulase production by *T*. *reesei* CICC2626 exposed to PEG8000 could be attributed to the overexpression of cellulase genes. Extracellular stress can be sensed by mechanoreceptors in the cytoskeleton, activating intracellular signaling pathways that transfer stress signals to the nucleus, resulting in changes in gene expression and enzyme synthesis (Ingber and Physiology 1997). As shown in Table 2, the transcriptional levels of the calcium signaling pathway–related genes (*plc-e*, *cam*, *can*, and *crz1*) were significantly upregulated after treatment with 1.5% PEG8000. Studies have suggested that different external stimuli, including metal ions (Schumacher et al. 2008), temperature (Chen et al. 2012), and ethanol (Araki et al. 2009), can activate the calcineurin-Crz1 signaling cascade. All these stresses cause an increase in cytosolic Ca^2+^ concentration. The increase in cytosolic Ca^2+^ levels led to the activation of calmodulin, which dephosphorylated its target proteins, such as Crz1. The dephosphorylated Crz1 was bonded to its target promoters in the nucleus to regulate gene expression (Manoli and Espeso 2019). Moreover, Chen et al. (2019) found that the phospholipase C-encoding gene *plc-e* were significantly upregulated during DMF-induced production of cellulase by *T*. *reesei* Rut-C30, suggesting that calcium signaling was involved in the expression of cellulase genes. To our knowledge, there are no former reports that show higher Ca^2+^ concentration in the medium promotes an increase in cellulose activities. The results of our previous study showed that the concentration of Ca^2+^ in the medium had little effect on *Bacillus subtilis* cellulase activities (Liu et al. 2022). These results demonstrate that the calcium signaling pathway may participate in PEG8000-induced cellulase overexpression in *T*. *reesei* CICC2626.

Ca^2+^ is an important intracellular signaling molecule that regulates primary and secondary metabolism in microorganisms (Zhang et al. 2013). Wang et al. (2020) reported that cellulase activity was positively correlated with increased cytosolic Ca^2+^ levels in *Ganoderma lucidum*. In this study, although PEG8000 induced a cytosolic Ca^2+^ burst, it was effectively blocked by Ca^2+^ inhibitors (LaCl_3_ and EGTA), resulting in decreased cellulase activity (Fig. 3). Crz1, a calcineurin-responsive zinc-finger transcription factor 1, has been found to play an essential role in calcium signal transduction in filamentous fungi (Sinha et al. 2021). Our work demonstrates that Ca^2+^-activated Crz1 participated in the regulation of cellulase gene expression following the PEG8000 treatment (Fig. 4). A similar result was found for *T. reesei* Rut-C30, in which cellulase activities in Δ*crz1* strain were reduced (Chen et al. 2018). In addition, Chen et al. (2019) demonstrated that intracellular Ca^2+^ plays an important role in the production of lignocellulolytic enzymes through the calcineurin-Crz1 signaling cascade under DMF induction in *T. reesei* Rut-C30. Therefore, it can be concluded that calcium signaling was involved in the expression regulation of cellulase genes in *T*. *reesei* CICC2626 exposed to PEG8000.

**Fig. 5:**
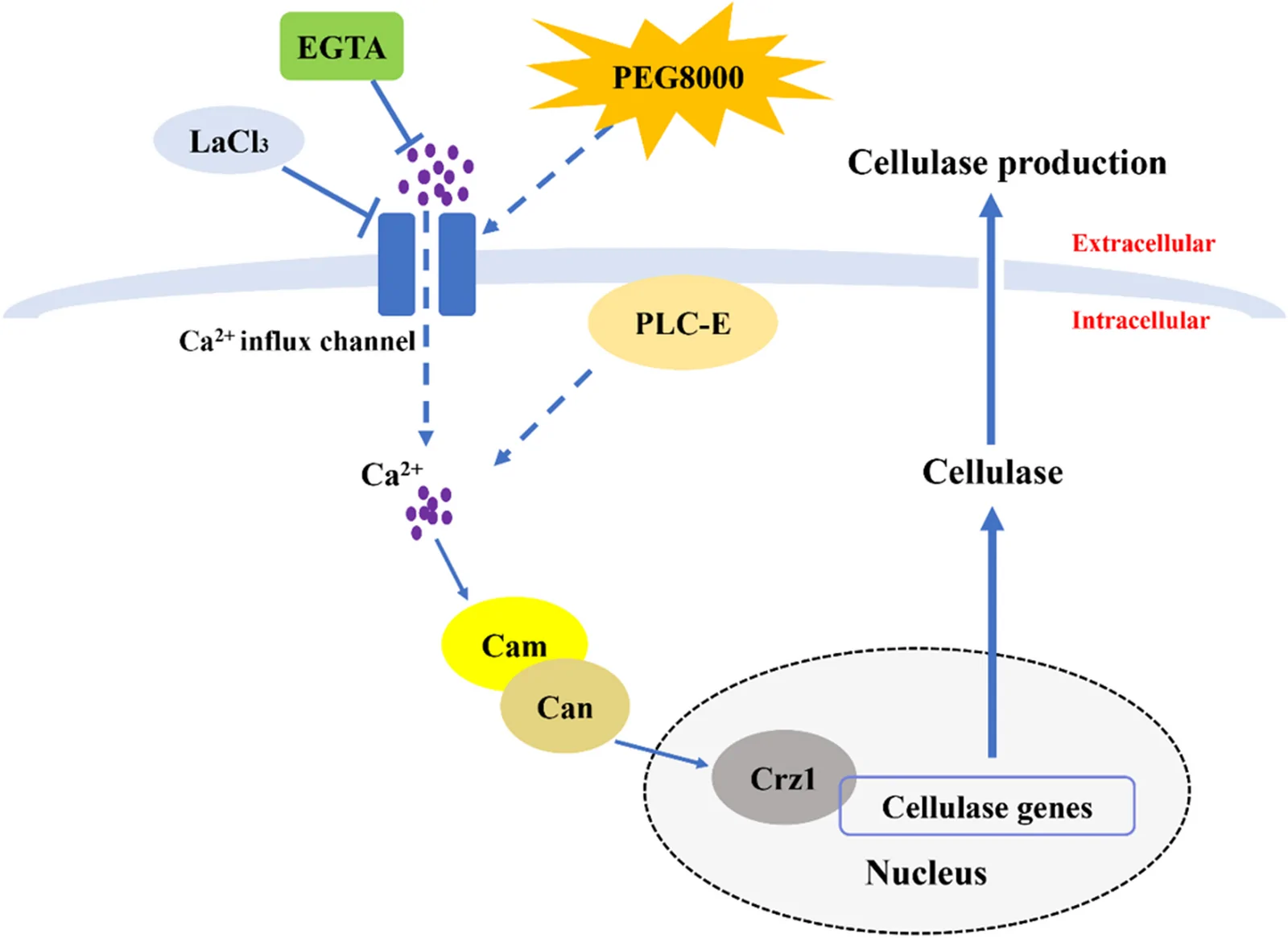
A possible mechanistic model by which calcium signaling regulates cellulase production in *T*. *reesei* CICC2626 induced by PEG8000. Solid arrows indicate data supported by this work; dashed arrows indicate undefined regulation

Based on the results of this study, a plausible mechanism by which calcium signaling regulated cellulase biosynthesis in *T. reesei* CICC2626 induced by PEG8000 was inferred (Fig. 5). The cytosolic Ca^2+^ level significantly increased after the PEG8000 treatment, which caused the transcriptional upregulation of calcium signaling pathway–related genes (*plc-e*, *cam*, *can*, and *crz1*) in *T. reesei*. The transcription factor Crz1 is then activated, thereby promoting the overexpression of cellulase genes (*egl1* and *cbh1*) in *T. reesei* treated with PEG8000. As a result, cellulase production by *T. reesei* is enhanced. The finding that cellulase activity decreased in the PEG8000 + LaCl_3_ and PEG8000 + EGTA groups and Δ*crz1* strain further supports this view. These results demonstrate that the calcium signaling pathway could regulate the overexpression of cellulase genes in *T. reesei*. Thus, a putative regulation mechanism on cellulase biosynthesis in *T. reesei* CICC2626 induced by PEG8000 was proposed.

## Supplementary Information

Below is the link to the electronic supplementary material.

Supplementary file1 (PDF 1066 KB)

Supplementary file2 (XLSX 2461 KB)

## Data Availability

The raw whole-transcriptome shotgun sequencing data have been deposited into the NCBI Sequence Read Archive (SRA) database under BioProject accession No. PRJNA761203.
