# Supplementary material for: Regulation of cellulase production via calcium signaling in Trichoderma reesei under PEG8000 stress
Source: Appl Microbiol Biotechnol. 2024 Jan 26;108:178. doi: 10.1007/s00253-023-12901-w (PMC10817842; doi:10.1007/s00253-023-12901-w)
Supplement: Supplementary file 1 [file 253_2023_12901_MOESM1_ESM.pdf]

SUPPLEMENTARY MATERIALS

for

Applied Microbiology and Biotechnology

Regulation of cellulase production via calcium signaling in *Trichoderma reesei* under PEG8000 stress

Shuai Liu [a,1], Lin Quan [a,1], Mei Yang [a], Dan Wang[b], Yong-Zhong Wang [a,*]

[a] Key Laboratory of Biorheological Science and Technology (Chongqing University), Ministry of Education, College of Bioengineering, Chongqing University, Chongqing 400030, China

[b]School of Chemistry and Chemical Engineering, Chongqing University, Chongqing 400044, China

* Corresponding author: Yong-Zhong Wang, E-mail: wangyzh@cqu.edu.cn (Y-Z. Wang)

[1] Shuai Liu and Lin Quan contributed equally to this work.

Supplementary Figures

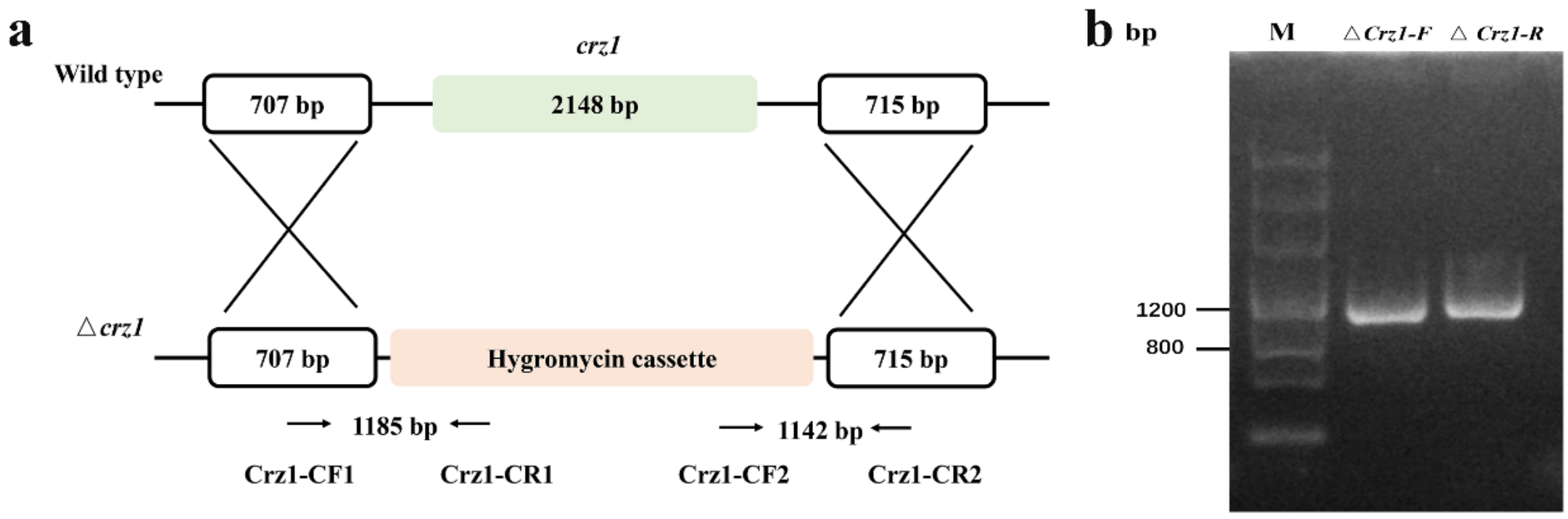

Fig. S1. Construction and verification of the Δ*crz1* mutant. **a** Schematic representation of the *crz1* locus from *T. reesei* CICC2626 and the Δ*crz1* mutant. The binding sites of primers on the genome of *T. reesei* CICC2626 and Δ*crz1*, and the expected sizes of the products in PCR verification are given. The region from +1 to +2148 bp relative to the translation start site of *crz1* (light green blue box) was replaced with hygromycin resistance expression cassette (Hygromycin, pink box). **b** PCR verification of the Δ*crz1* mutant. Lane M, DNA molecular mass marker. PCR amplification results of Δ*Crz1*-F were obtained using Crz1-CF1 with Crz1-CR1 and Δ*Crz1*-R were obtained using Crz1-CF2 with Crz1-CR2 (Table S1).

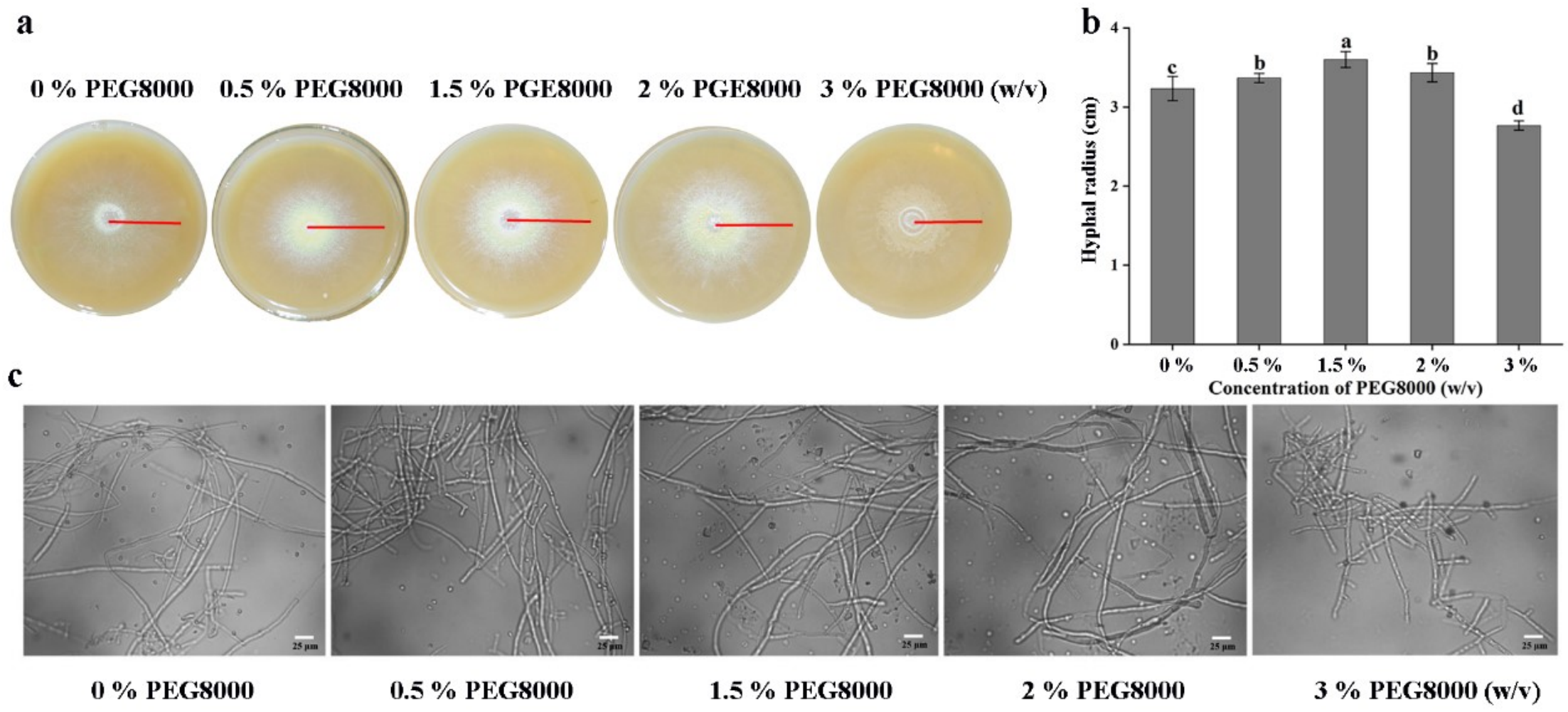

Fig. S2. Hyphal growth and morphology of *T. reesei* CICC2626. PEG8000 was added at final concentrations of 0 % (control), 0.5 %, 1.5 %, 2 % or 3 % (w/v). The corresponding osmotic pressure was 203, 206, 229, 236, and 268 mOsm/kg. **a** Hyphal growth of *T. reesei* CICC2626 on YPD plates. **b** Colony radius of *T. reesei* CICC2626 on YPD plates. The red line represents mycelium morphologies throughout the 4th day of culture. **c** Hyphal morphology of *T. reesei* CICC2626 was imaged using an inverted fluorescent microscope (Leica, Wetzlar, Germany) after 4 days of culture. Different letters in b. indicate significant differences between the columns ($p < 0.05$, Duncan's multiple-range test).

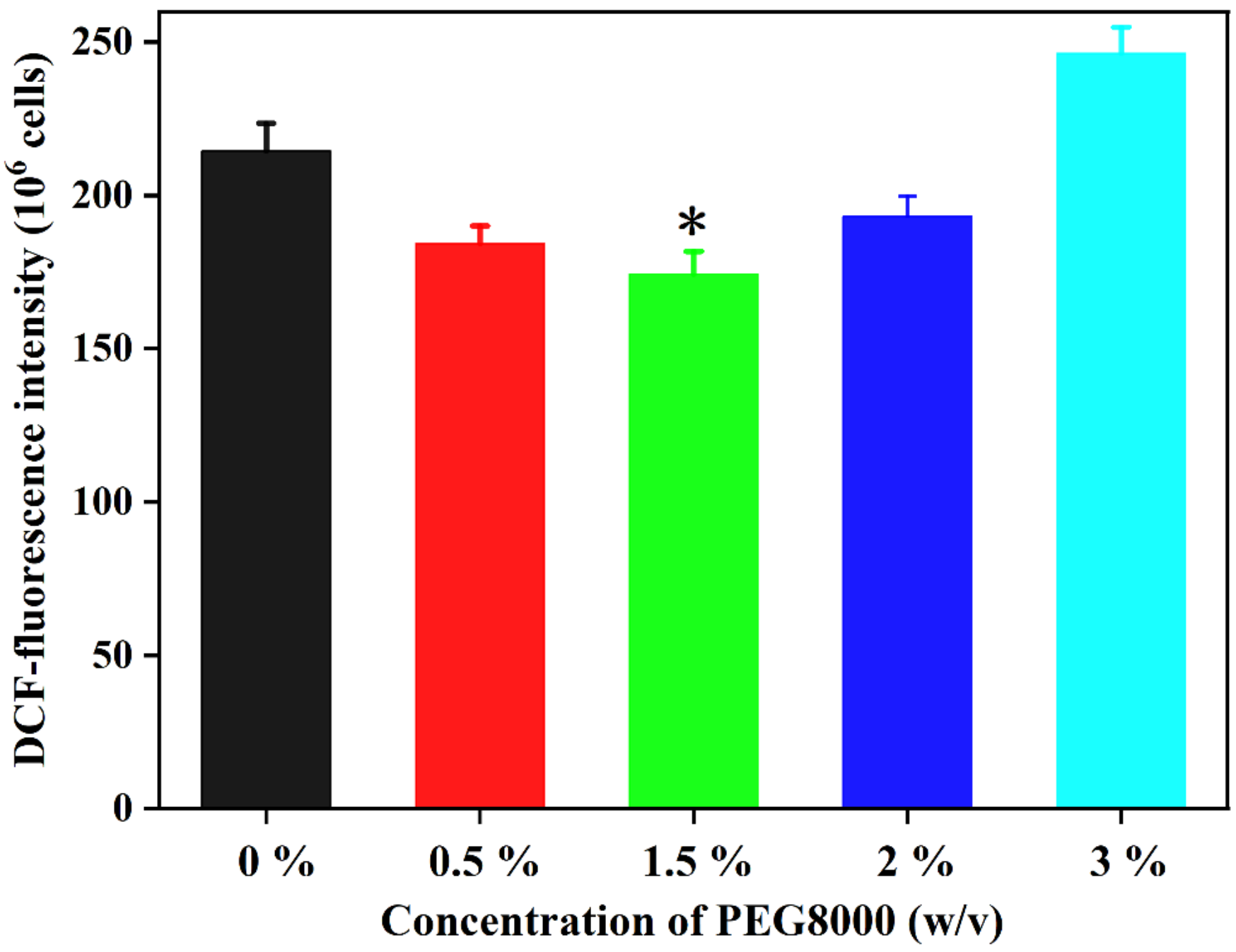

Fig. S3. Effects of different concentrations of PEG8000 at the final concentrations of 0, 0.5, 1.5, 2, and 3 % (w/v) on ROS concentration of *T. reesei* CICC2626. The seed solution of *T. reesei* CICC2626 was cultured in YPD medium for 24 h; then transferred to fresh MM medium containing 2 % (w/v) Avicel with 0 to 3 % PEG8000, and then cultivated for further 4 d. Values are the mean ± SD of the results from three independent experiments. Asterisks indicate significant differences (*p < 0.05, **p < 0.01, Student's t test).

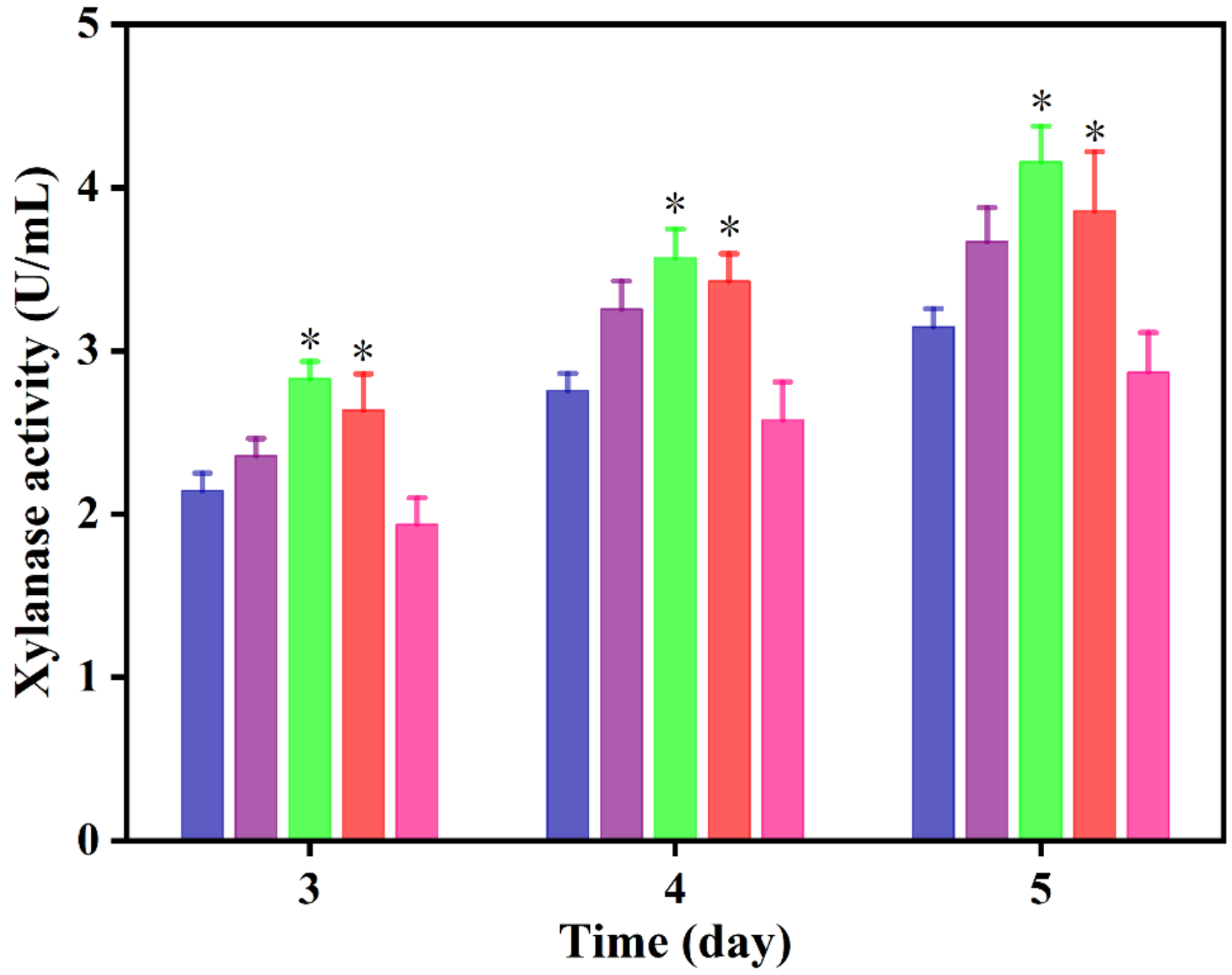

Fig. S4. Effects of different concentrations of PEG8000 at the final concentrations of 0, 0.5, 1.5, 2, and 3 % (w/v) on xylanase activity of *T. reesei* CICC2626. The seed solution of *T. reesei* CICC2626 was cultured in YPD medium for 24 h; then transferred to fresh MM medium containing 2 % (w/v) Avicel with 0 to 3 % PEG8000, and then cultivated for further 36 to 120 h. Blue bar, addition of 0 % (w/v) PEG8000; purple bar, addition of 0.5 % (w/v) PEG8000; green bar, addition of 1.5 % (w/v) PEG8000; red bar, addition of 2 % (w/v) PEG8000; pink bar, addition of 3 % (w/v) PEG8000. Values are the mean ± SD of the results from three independent experiments. Asterisks indicate significant differences (*$p < 0.05$, **$p < 0.01$, Student's *t* test).

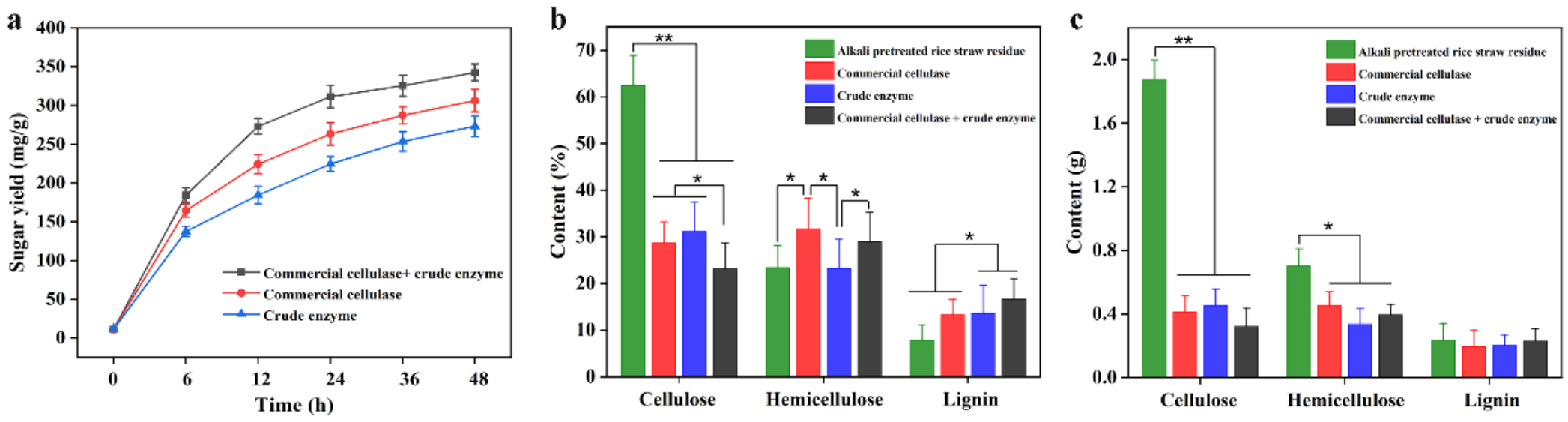

Fig. S5. Synergistic hydrolysis of pretreated rice straw by crude enzyme and commercial cellulase from 0 % PEG8000 supplement culture. a The concentration of reducing sugar after hydrolysis. b-c The percentage and weight of cellulose, hemicellulose and lignin in alkali-pretreated rice straw residues after hydrolysis. The hydrolysis reaction was carried out with 15 % (w/v) alkali-pretreated rice straw in a 20 mL system. After the hydrolysis reaction, the treated residue yield of groups commercial cellulase, crude enzyme and commercial cellulase + crude enzyme from rice straw was 1.42, 1.46 and 1.38 g respectively. Values are the mean ± SD of the results from three independent experiments. Asterisks indicate significant differences (*p < 0.05, Student's t test).

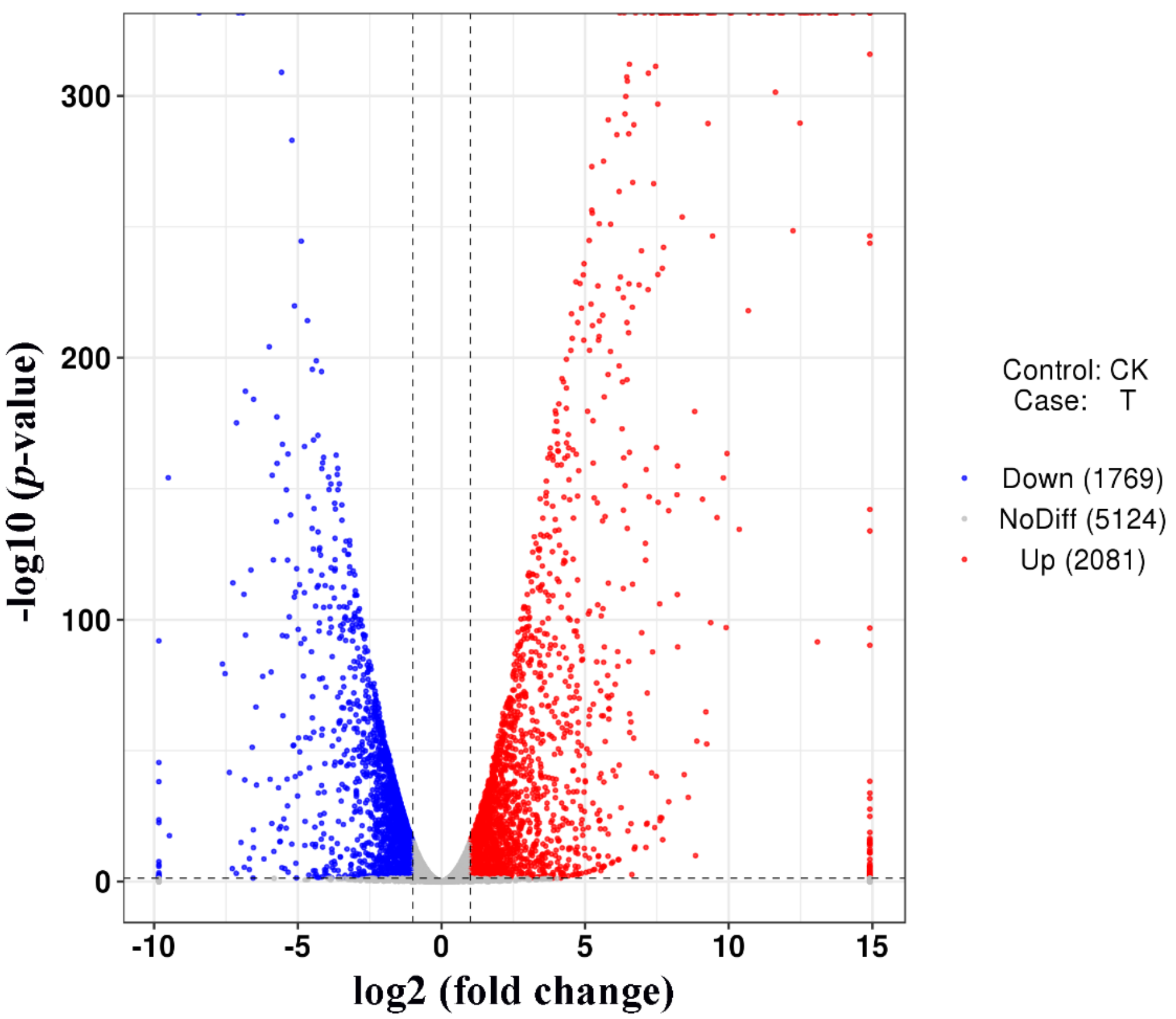

Fig. S6. Volcano plot analysis of up- and down-regulated genes of strains treated with 0 or 1.5 % (w/v) PEG8000. CK, control group; T, PEG8000 group. Red dots indicate significantly up-regulated genes; green dots indicate significantly down-regulated genes; gray dots indicate non-significantly different gene expression.

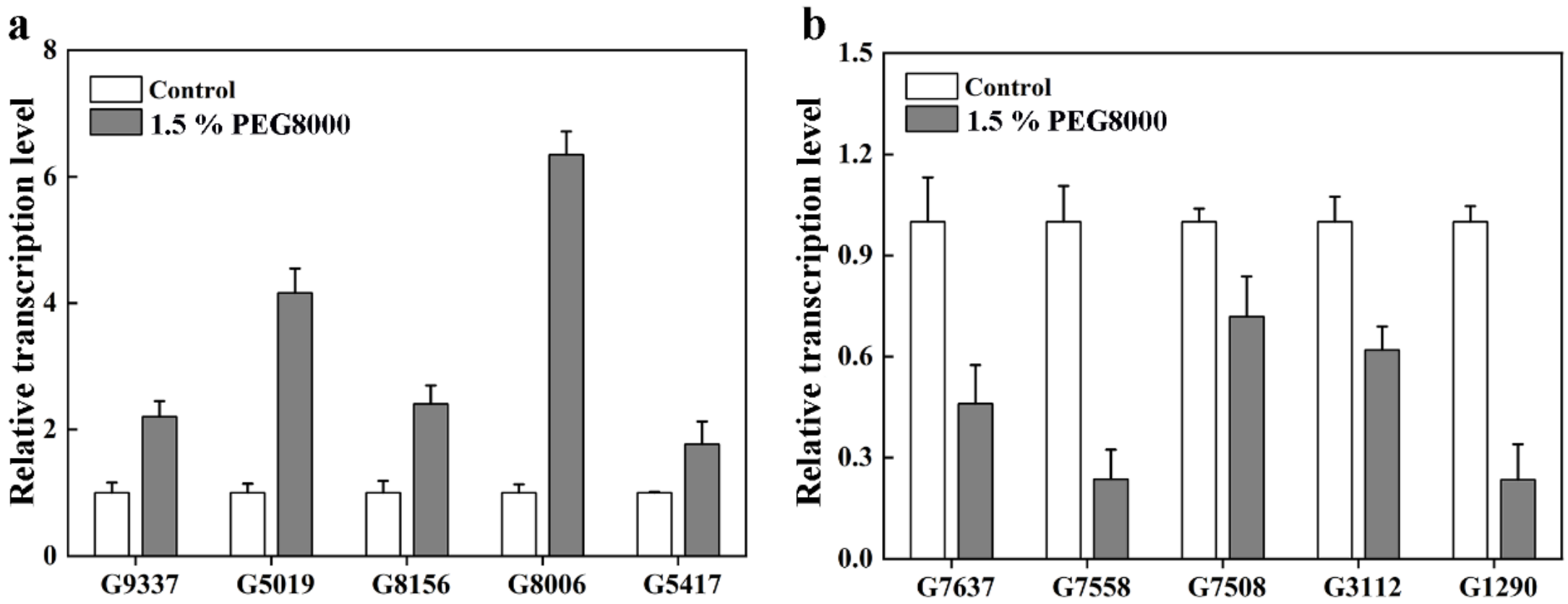

Fig. S7. RT-qPCR validation of ten differentially expressed genes (DEGs) at the mRNA level. Expression of five representative up-regulated genes (**a**) and five representative down-regulated genes (**b**) was determined to confirm the transcriptome data. CK, control group; T, PEG8000 group. The $2^{-\triangle\triangle CT}$ values were calculated from quadruplicate runs of two independent experiments.

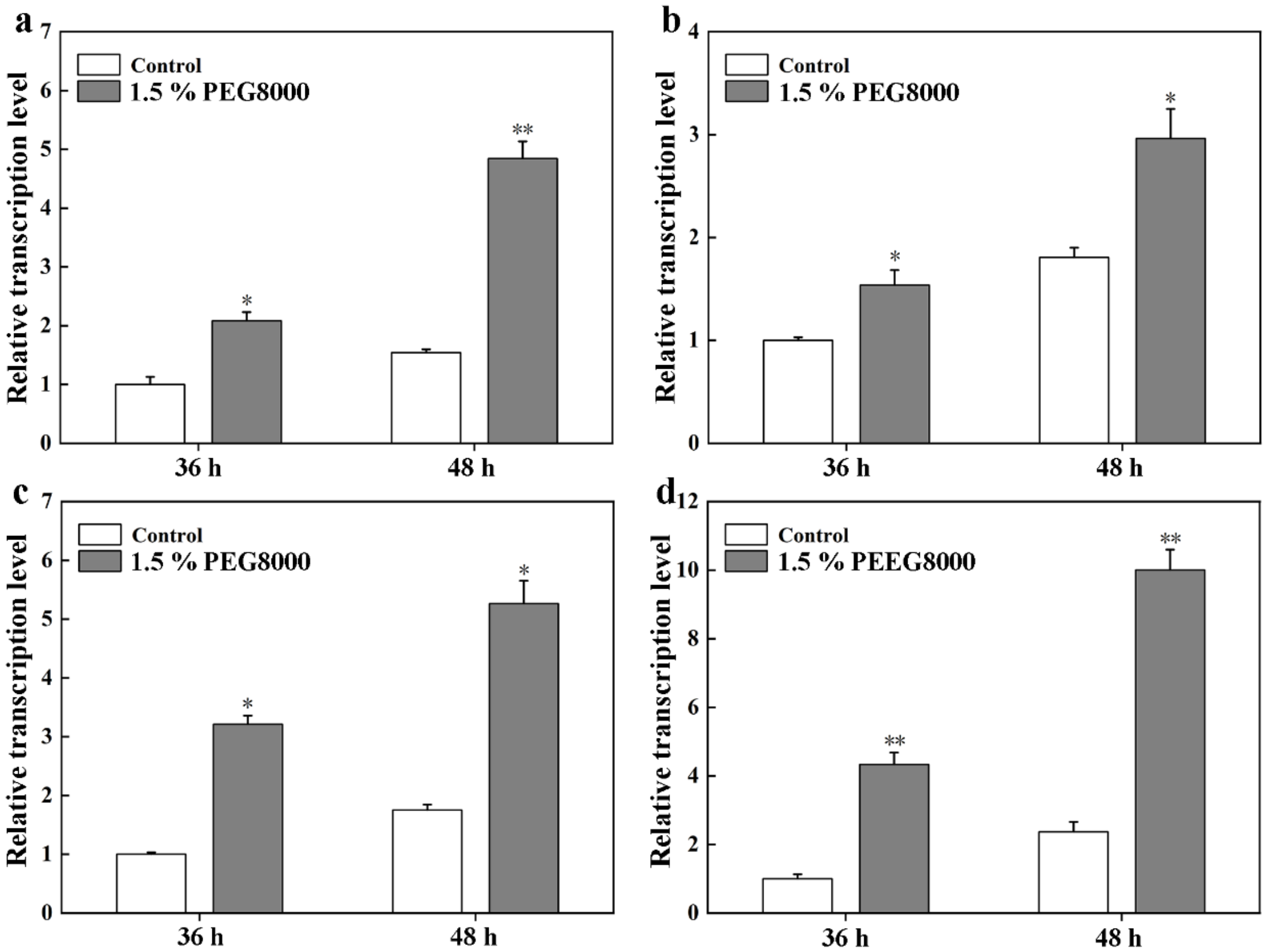

Fig. S8. The effect of PEG8000-induced osmotic stress on the expression levels of Ca^{2+} signaling-related genes *plc-e* (**a**), *cam* (**b**), *can* (**c**) and *crz1* (**d**) in *T. reesei* CICC2626. Values are the mean ± SD of the results from three independent experiments. Asterisks indicate significant differences from untreated strains (*$p < 0.05$, ** $p < 0.01$, Student's *t* test).

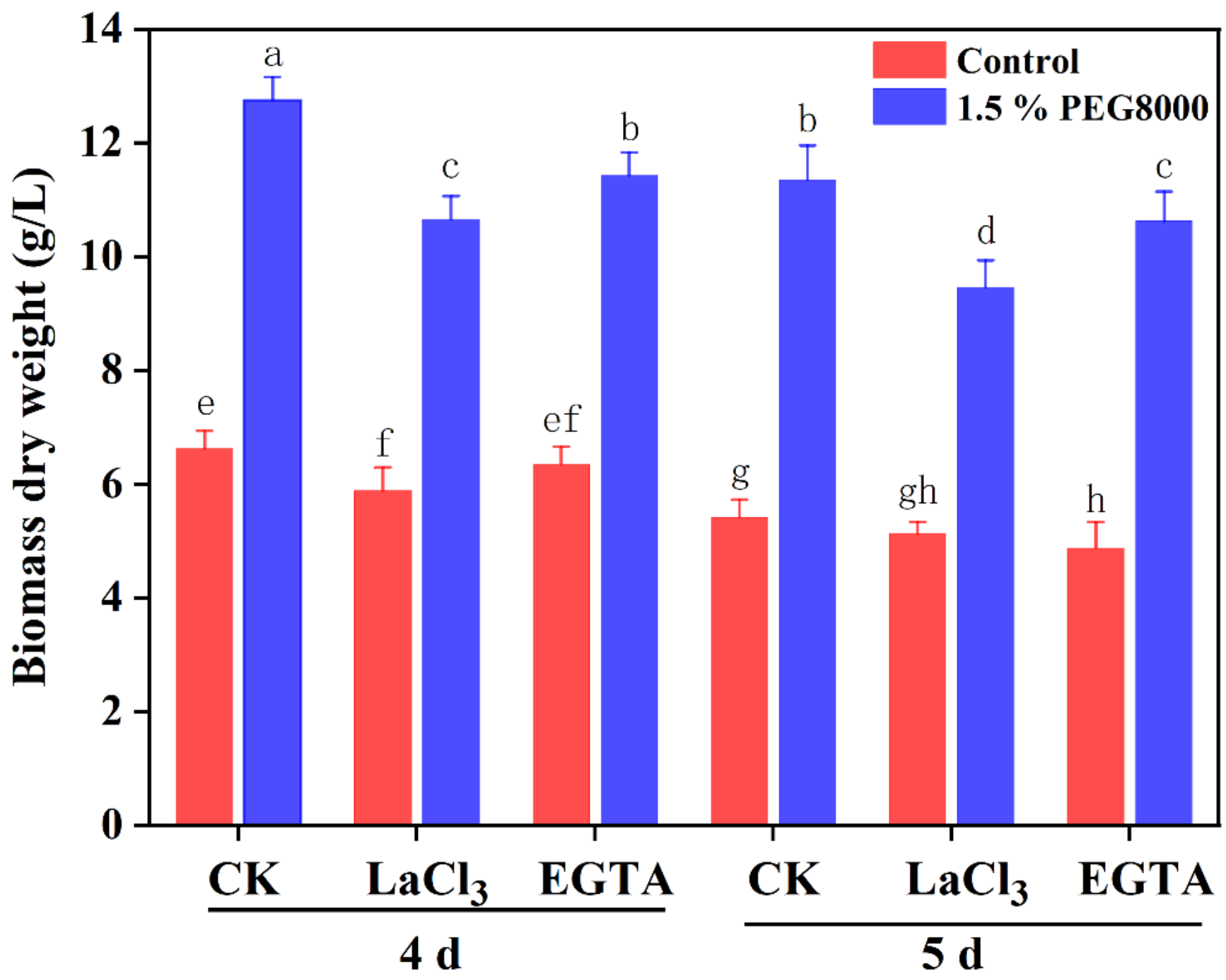

Fig. S9. Effects of Ca^{2+} inhibitors on the biomass of *T. reesei* CICC2626. *T. reesei* CICC2626 was cultured in YPD medium for 24 h, transferred to fresh MM containing 2 % (w/v) Avicel with 0 or 1.5 % (w/v) PEG8000 and 0 or 10 mM $LaCl_3$/EGTA, and cultivated further for 4 to 5 d. Values are mean ± standard deviation (n = 3). Different letters indicate significant differences between the columns ($p < 0.05$, Duncan's multiple-range test).

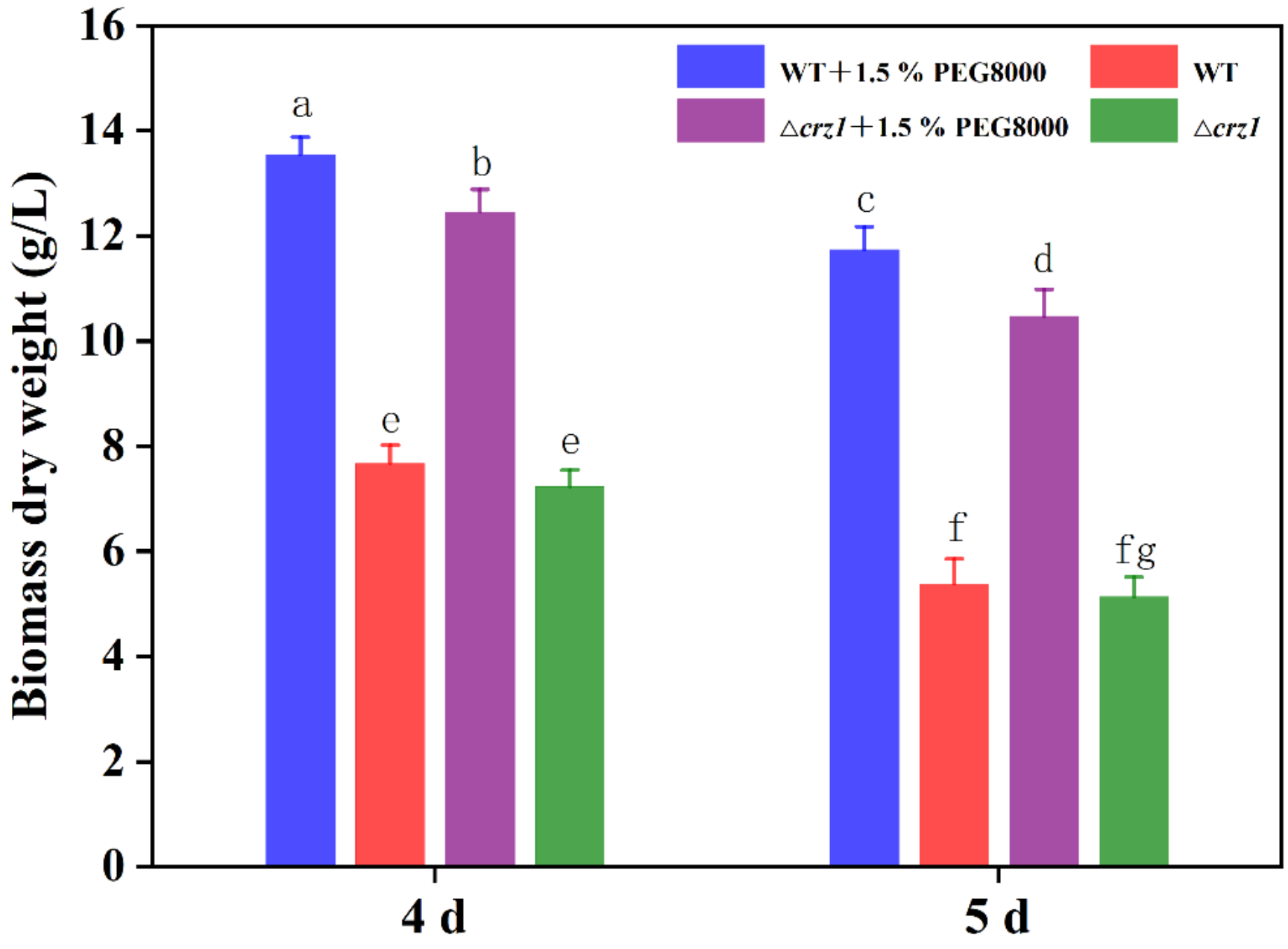

Fig. S10. Effect of *crz1* on the biomass of wild type and Δ*crz1* strains. Wild type and Δ*crz1* strains were cultured in YPD medium for 24 h, transferred to fresh MM containing 2 % (w/v) Avicel with 0 % or 1.5 % (w/v) PEG8000, and cultivated further for 4 to 5 d. Values are mean ± standard deviation (n = 3). Different letters indicate significant differences between the columns ($p < 0.05$, Duncan's multiple-range test).
